# Heparan sulfate proteoglycans serve as alternative receptors for low affinity LCMV variants

**DOI:** 10.1371/journal.ppat.1009996

**Published:** 2021-10-14

**Authors:** André Volland, Michael Lohmüller, Emmanuel Heilmann, Janine Kimpel, Sebastian Herzog, Dorothee von Laer

**Affiliations:** 1 Institute of Virology, Medical University of Innsbruck, Innsbruck, Austria; 2 Division of Developmental Immunology, Medical University of Innsbruck, Innsbruck, Austria; University of Alabama at Birmingham, UNITED STATES

## Abstract

Members of the Old World Arenaviruses primarily utilize α-dystroglycan (α-DAG1) as a cellular receptor for infection. Mutations within the glycoprotein (GP) of lymphocytic choriomeningitis virus (LCMV) reduce or abrogate the binding affinity to α-DAG1 and thus influence viral persistence, kinetics, and cell tropism. The observation that α-DAG1 deficient cells are still highly susceptible to low affinity variants, suggests the use of an alternative receptor(s). In this study, we used a genome-wide CRISPR Cas9 knockout screen in DAG1 deficient 293T cells to identify host factors involved in α-DAG1-independent LCMV infection. By challenging cells with vesicular stomatitis virus (VSV), pseudotyped with the GP of LCMV WE HPI (VSV-GP), we identified the heparan sulfate (HS) biosynthesis pathway as an important host factor for low affinity LCMV infection. These results were confirmed by a genetic approach targeting EXTL3, a key factor in the HS biosynthesis pathway, as well as by enzymatic and chemical methods. Interestingly, a single point mutation within GP1 (S153F or Y155H) of WE HPI is sufficient for the switch from DAG1 to HS binding. Furthermore, we established a simple and reliable virus-binding assay, using directly labelled VSV-GP by intramolecular fusion of VSV-P and mWasabi, demonstrating the importance of HS for virus attachment but not entry in Burkitt lymphoma cells after reconstitution of HS expression. Collectively, our study highlights the essential role of HS for low affinity LCMV infection in contrast to their high affinity counterparts. Residual LCMV infection in double knockouts indicate the use of (a) still unknown entry receptor(s).

## Author summary

Lymphocytic choriomeningitis virus (LCMV) contributed to the fundamental understanding of immunological processes due to characteristics such as persistent and immunosuppressive infection. However, not all strains of LCMV share the same traits. Differences in tissue tropism and the course of disease led to intensive characterization of distinct LCMV variants. Point mutations within the glycoprotein of LCMV were identified that reduce or abrogate the binding affinity to its host receptor α-Dystroglycan (α-DAG1), leading to the classification of low and high affinity LCMV variants. α-DAG1-independent infection and altered tissue tropism suggested the use of an alternative, unknown receptor by low affinity LCMV variants. Applying a genome-wide knockout screen and comparing different LCMV strains by genetic, enzymatic, and chemical approaches, we identified heparan sulfate proteoglycans (HSPG) as alternative receptors favoured by low affinity LCMV variants. These findings improve the understanding of receptor usage by different LCMV variants and explain their distinct characteristics. Furthermore, residual LCMV infection of double knockout cells indicate a major role of α-DAG1 and HSPG as attachment factors for high and low affinity LCMV variants, respectively, as well as the use of (a) still unknown entry receptor(s).

## Introduction

Arenaviruses are enveloped RNA viruses with a bi-segmented ambisense genome, consisting of a large segment that encodes the viral RNA-dependent RNA polymerase (L) and the matrix protein (Z) as well as a small segment encoding the viral nucleocapsid protein (N) and the glycoprotein precursor (GPC). Similar to other enveloped viruses, GPC is further processed by cellular proteases to generate functional GP1/GP2, with its host cell binding region located in GP1 and the fusogenic site in GP2 [1].

Several Old World Arenaviruses including LCMV and members of the clade C New World Arenaviruses bind to the ubiquitously expressed cellular receptor Dystroglycan (DAG1) [2,3]. DAG1 is expressed as a precursor polypeptide and posttranslationally cleaved in two non-covalently bound subunits termed α-DAG1 and β-DAG1 [4]. Binding of extracellular matrix (ECM) proteins such as laminin, agrin or perlecan to extracellular α-DAG1 and interaction of membrane-spanning β-DAG1 with cytoskeletal proteins, provides a direct link between the ECM and the cytoskeleton [5]. Proper O-glycosylation of extracellular α-DAG1 is essential for its functionality. Defects in glycosyltransferases are not only linked to several forms of muscular dystrophy [6] but also to the loss of receptor binding of Lassa virus (LASV) and LCMV [7,8]. Thus, ligand and arenavirus binding to α-DAG1 relies on like-acetylglucosaminyl-transferase (LARGE)-dependent modifications at Thr-317 and -319 within the mucin-like domain [9] by synthesis and elongation of matriglycan [10]. However, not all LCMV variants are dependent on functional DAG1 for virus binding and infection. Single point mutations within the GP1 domain at positions S153F, Y155H, and L260F [11–13] are described to alter the binding affinity to α-DAG1 and facilitate binding to an alternative, still unknown receptor. This resulted in a classification into low (e.g. WE2.2, Arm 53b, HPI WT) and high affinity (e.g. Arm Cl13, WE54) LCMV variants. Interestingly, DAG1 knockout cells, although highly reduced, are still susceptible to high affinity LCMV infection [13].

Members of the Tyro3/Axl/Mer (TAM) family as well as DC-SIGN and LSECtin were identified in a cDNA library screen as alternative receptors for LASV [14] and LCMV [15]. The infection of lentiviruses pseudotyped with the GP of low or high affinity LCMV variants was equally enhanced in Jurkat cells expressing Axl, Tyro3, DC-SIGN or LSECtin, indicating that these are not alternative receptors exclusively used by low affinity variants. In addition, the role of Axl is controversial, because no effect on LCMV infection was observed in an Axl knockout mouse model [16]. In contrast, phosphatidylserine (PtdSer)-mediated and DAG1-independent entry by the PtdSer receptors Axl and TIM-1 was shown for the closely related LASV [1,17]. PtdSer-mediated virus entry, also known as apoptotic mimicry, is GP-independent and exploited as an alternative entry pathway by various enveloped viruses [18,19]. Since LASV GP pseudotyped LCMV or VSV virions were used to study Axl and TIM-1 mediated entry, it is most likely that LCMV itself can utilize this pathway in the absence of its preferred host receptor. Nevertheless, PtdSer-mediated virus uptake cannot explain differences between low and high affinity LCMV variants in DAG1 deficient cells. Interestingly, mutations within the GP1 domain not only affect the cell tropism and virus kinetic, but also influence virus persistence. Variants with a high affinity for DAG1 are classified as persistent and immunosuppressive pathogens, whereas low affinity binders are non-immunosuppressive and are efficiently cleared by the hosts immune system [13]. Successful pseudotyping of several viral vectors such as retroviruses, lentiviruses or vesicular stomatitis virus (VSV) with LCMV GP were described previously [20–23]. This allows for easy and rapid analysis of the unique cell tropism mediated by different LCMV GP variants.

We found that DAG1 expression in several cell lines did not correlate with tumour cell line and tumour graft susceptibility using the GP of low affinity variant HPI WT (Y155H) in an oncolytic VSV-based platform (VSV-GP) [24]. This underlines the assumption that low affinity LCMV GP does not need DAG1 in order to mediate cell entry and uses one or several alternative receptor(s). Therefore, we aimed to identify the alternative receptor(s) for low affinity LCMV variants. For this, we used a genome-wide CRISPR Cas9 knockout screen, which has already been successfully used to identify host factors for several viruses [25–30]. Significant enrichment of knockouts involved in the heparan sulfate (HS) biosynthesis pathway correlated with reduced susceptibility against VSV-GP HPI WT in 293T DAG1 deficient cells. In this manuscript, we demonstrate the importance of HS proteoglycans (HSPG) as alternative receptors by genetic, enzymatic and chemical approaches in different cell lines.

## Results

### A genome-wide CRISPR Cas9 knockout screen identifies host factors involved in DAG1-independent LCMV infection

**Fig 1:**
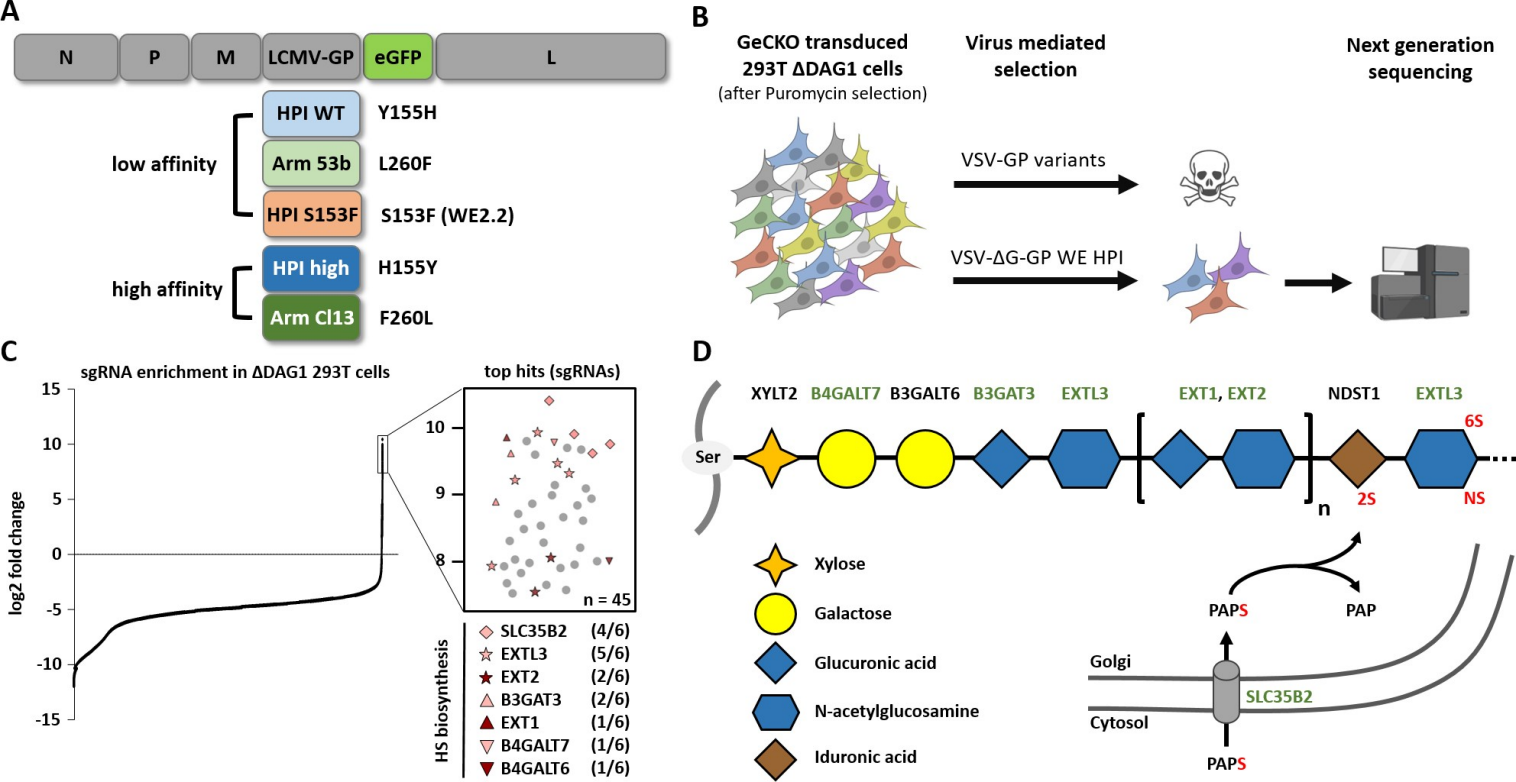
Genome-wide CRISPR Cas9 knockout screen identifies host factors import for LCMV infection in the absence of DAG1. (A) Schematic overview of the genome of replication competent VSV-based reporter systems pseudotyped with high or low affinity LCMV GP variants and *eGFP* as a transgene. (B) Schematic overview of CRISPR Cas9-mediated knockout screen in 293T Δ*DAG1* cells with replication competent or incompetent high and low affinity VSV-GP variants. Created with BioRender.com. (C) Enrichment of sgRNA expression after low affinity VSV-ΔG-GP (HPI WT) selection compared to an untreated control (left). Representation of top hits and number of significantly enriched sgRNAs (out of 6) per gene (right; n = 45) and (D) their roles in the heparan sulfate biosynthesis pathway. Enriched genes from the genome-wide CRISPR Cas9 screen are marked in green.

To identify host factors involved in DAG1-independent LCMV infection, we performed three rounds of pooled genome-wide CRISPR Cas9 knockout screens in 293T DAG1 knockout cells (293T Δ*DAG1*). For this, 2x10^8^ 293T Δ*DAG1* cells were transduced with the lentiviral GeCKO Library at an MOI of 0.3 [31]. Ten days after puromycin selection, 3x10^8^ cells were challenged with replication competent (MOI 1) or incompetent (single round infectious, MOI 10) vesicular stomatitis virus (VSV), pseudotyped with the glycoprotein (GP) of LCMV (VSV-GP or VSV-ΔG-GP). In this study, two high and three low affinity LCMV-GP variants were used: HPI WT (Y155H), WE HPI S153F (WE2.2) and Arm 53b (L260F), all three carrying low affinity mutations, and HPI high (H155Y) and Arm Cl13 (260L) which are classified as high affinity variants (**Fig 1A and 1B and S1 Table**). Parallel to virus-mediated selection, uninfected cells were further cultured to later serve as an untreated control and reference for bioinformatic analysis.

Selection of completely resistant cells after infection with replication competent high or low affinity VSV-GP variants failed. There are several potential explanations, such as the use of multiple entry receptors, a family of receptors with high similarity between members or non-specific entry pathways as described for other viruses. At cellular level, this may indicate an essential role of the alternative receptor for cell survival, attachment or cell growth. To achieve infection by a defined MOI and thus avoid unspecific uptake due to excessive virus progeny, we decided to use replication incompetent VSV-ΔG-GP WE HPI for selection. This strategy allowed isolation of cells that were not completely resistant but showed highly reduced susceptibility to VSV-GP infection.

After three rounds of infection, the genomic DNA was isolated and deep-sequenced. From three independent selection experiments most hits were involved in the heparan sulfate biosynthesis pathway such as synthesis of glycosaminoglycan (GAG) (*B4GALT7*, *B3GALT6* and *B3GAT3*), the elongation of heparan sulfate (HS) chains (*EXTL3*, *EXT1* and *EXT2*) and *SLC35B2* encoding the Golgi-resident transporter of the universal sulfate donor 3’-phosphoadenosine-5’-phosphosulfate (PAPS) (**Fig 1C and 1D and S6 Table**). Other enriched hits associated with HS expression were the component of oligomeric golgi complex 1 (*COG1*) and transmembrane protein 165 (*TMEM165*). HS expression unrelated but enriched genes included UNC-50 inner nuclear membrane RNA binding protein (*UNC50*), ADP ribosylation factor related protein 1 (*ARFRP1*) and mitochondrial elongation factor 1 (*MIEF1*) (for detailed information see **S6 Table**). These results indicate an important role of HS as a host factor involved in low affinity VSV-GP infection.

### Lack of cell surface HS inhibits infection with LCMV variants

To validate the role of HS for LCMV infection, knockout cells (293T and L929) were generated using the CRISPR Cas9 system. We designed two human and two murine guide RNAs (gRNAs) targeting the *EXTL3* gene, encoding for a key enzyme in the elongation process of HS chains [32] and, along with *SLC35B2*, was highly enriched in the genome-wide CRISPR Cas9 knockout screen (**Fig 1C**). To further analyse the role of HS in DAG1-independent LCMV infection, we designed human double knockout cells lacking the expression of both, DAG1 and EXTL3 (293T Δ*DAG1 EXTL3*). The natural absence of functional DAG1 makes the murine fibroblast cell line L929 a perfect model for LCMV-GP mediated DAG1-independent infection analysis and validation of results obtained with 293T cells. Moreover, EXTL3 knockout in L929 cells therefore represents a double knockout of DAG1 and HS expression. All cell clones were generated by single cell dilution after CRISPR Cas9-mediated knockout.

**Fig 2:**
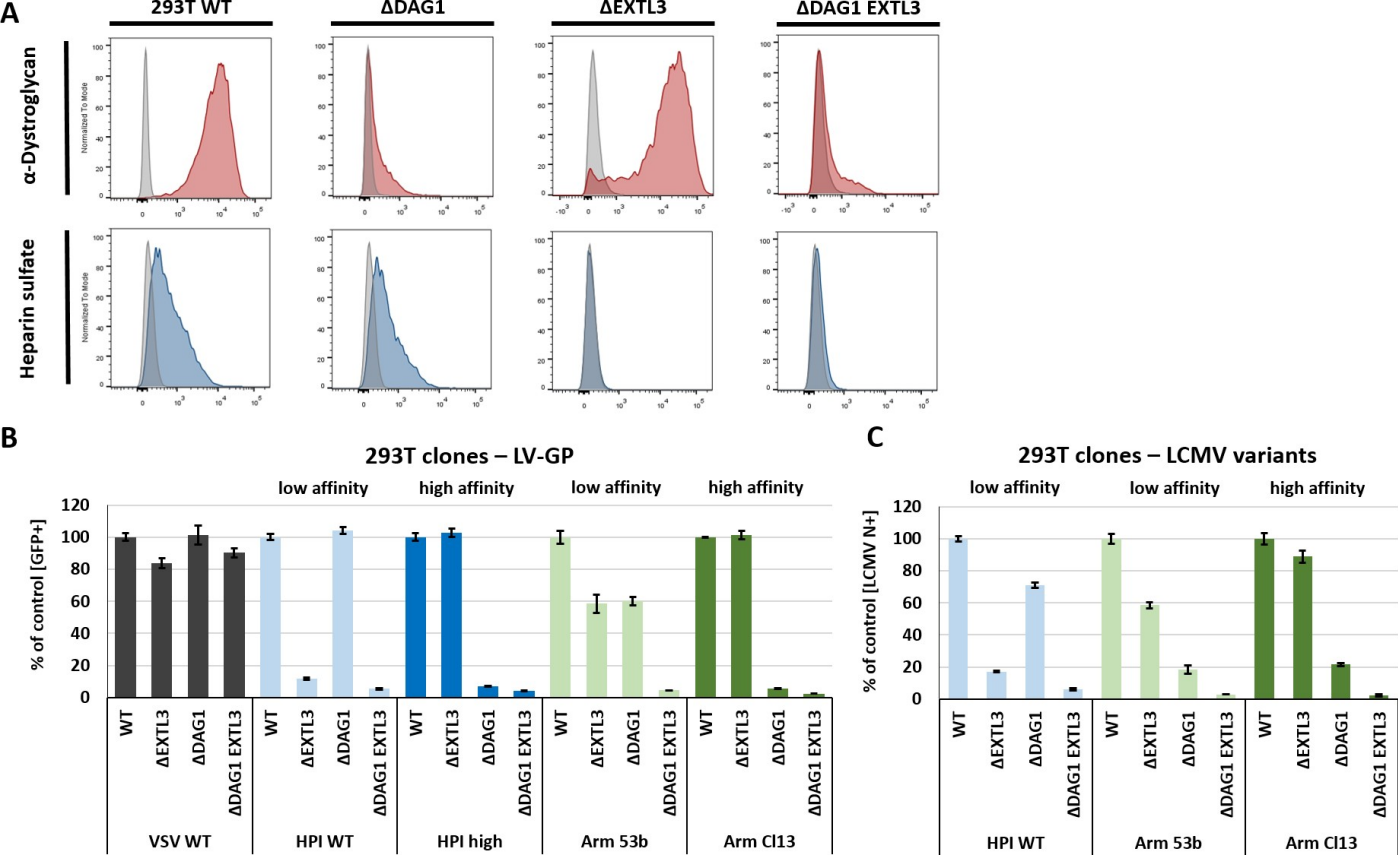
Lack of HS after EXTL3 knockout reduces LCMV infection. (A) Flow cytometry analysis of Dystroglycan-1 (IIH6; red) and Heparan sulfate (10E4; blue) expression in 293T WT, Δ*DAG1*, Δ*EXTL3*, and Δ*DAG1 EXTL3* cells generated by CRISPR Cas9-mediated knockout. (B) Infection assay using a lentiviral (LV) reporter system encoding eGFP. LVs were pseudotyped with VSV-G, as a control, and low (HPI WT Y155H or Arm 53b L260F) or high (HPI high H155Y or Arm Cl13 260L) affinity LCMV GP variants. Cells were transduced with an MOI of 1 (determined for 293T WT) and eGFP signal was measured 72 h later by flow cytometry. (C) Infection assay with low (WE HPI or Arm 53b) and high (Arm Cl13) affinity variants of LCMV in different knockout variants of 293T cells. The cells were infected with an MOI of 1 (determined by semi-functional quantitative flow cytometry assay[55] for 293T WT cells) for 2 h at 37°C and 16 h p.i. quantified by flow cytometry via LCMV N-staining. Shown are the means ± SD of three replicates.

Single and double knockout clones for *DAG1* and/or *EXTL3* were confirmed by flow cytometry analysis (**Figs 2A and S2A**) and sequencing (not shown). Infection efficacy with VSV-G pseudotyped lentivirus (LV) as a control showed no major differences between the knockout variants and the parental cells (**Fig 2B**). In contrast, both knockouts had an effect on infection of LCMV GP pseudotyped LV. The knockout of *EXTL3* highly reduced susceptibility to low affinity LV-GP HPI WT (Y155H), while the lack of DAG1 had no effect. Vice versa, Δ*DAG1* cells were poorly infected with both high affinity variants LV-GP HPI high (H155Y) and LV-GP Arm Cl13 (260L), whereas no differences were observed in Δ*EXTL3* cells. Low affinity LV-GP Arm 53b (L260F) showed reduced infection of about ~ 40% in both Δ*EXTL3* and Δ*DAG1* cells compared to WT cells. Infection via high and low affinity GP was further reduced to a very low level in double knockout 293T cells (Δ*DAG1 EXTL3*) compared to the single knockout cells (**Figs 2B and S2B**). Multi-step growth kinetics performed with VSV WT and low or high affinity VSV-GP variants over 48 h in 293T WT and knockout variants are in line with the data generated with pseudotyped lentiviruses. Single knockout of EXTL3 or DAG1 resulted in reduced virus titre for low or high affinity VSV GP variants, respectively. This reduction was even stronger in double knockout (Δ*DAG1 EXTL3)* cells for all variants (**S1 Fig**). These results could be confirmed by infection studies with replication competent LCMV (HPI WT, Arm 53b, and Arm Cl13) (**Fig 2C**). Thus, direct targeting of the key enzyme EXTL3 of the HS biosynthesis pathway proved the importance of HS as a host factor involved in viral infection of low affinity variants even in the presence of DAG1 and for high affinity variants in the absence of functional DAG1.

**Fig 3:**
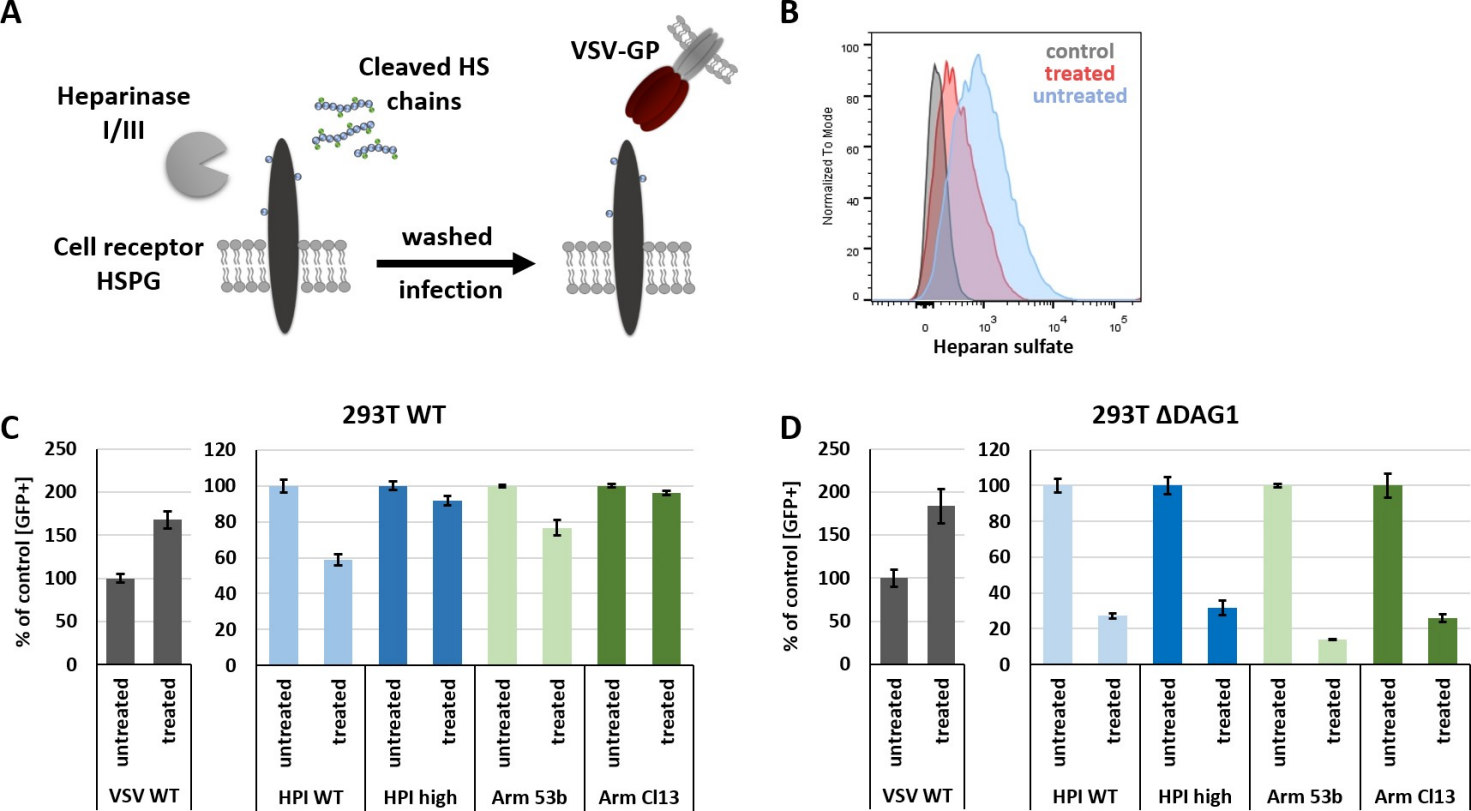
Enzymatic digestion of HS by Heparinase I/III inhibits LCMV GP mediated virus infection. (A) Schematic overview of Heparinase I/III digestion of HS chains located on HSPGs on the cell surface. Cells were pre-treated for 2 h at 37°C with 1 Unit Heparinase I/III. Subsequently, cells were washed and infected with VSV WT or VSV-GP variants (MOI 1) for 1 h at 37°C in complete growth medium. (B) Flow cytometry analysis of cell surface HS of Heparinase I/III treated and untreated 293T WT cells. Infection of Heparinase I/III treated (C) 293T WT and (D) Δ*DAG1* cells with VSV WT and high or low affinity VSV-GP variants. Infection was quantified by flow cytometry measuring eGFP signal 15 h p.i. Shown are the means ± SD of three replicates.

Next, we treated WT (293T and L929) and 293T Δ*DAG1* cells with Heparinase I/III to remove HS from the cell surface. After treatment with 1 Unit/ml Heparinase I/III for 2 h at 37°C the cells were washed and infected with VSV WT or VSV-GP variants for 1 h at 37°C. HS cleavage should result in reduced LCMV binding and infection comparable to a double knockout (Δ*DAG1 EXTL3*) (**Fig 3A**).

Enzymatic digestion of HS by Heparinase I/III was confirmed by flow cytometry analysis (**Figs 3B and S2C**). Treatment of 293T WT and Δ*DAG1* cells with Heparinase I/III highly increased infection with control virus VSV-G, as previously observed [33]. In contrast, infection with low affinity VSV-GP variants was reduced by 20–40% in 293T WT (**Fig 3C**) and by 75–85% *ΔDAG1* cells (**Figs 3D and S2D**), respectively. Infection with high affinity VSV-GP variants was only inhibited in *ΔDAG1* cells. These results are weaker but consistent with the reduction observed for 293T Δ*EXTL3* and 293T Δ*DAG1 EXTL3* infection (**Fig 2B and 2C**), which may be explained by incomplete digestion of HS after Heparinase I/III treatment (**Figs 3B and S1C**). These results further confirm a key role of HS in high and low affinity VSV-GP infection.

### Soluble heparin and protamine sulfate inhibit infection of VSV-GP variants

We next assessed the ability of soluble heparin to inhibit LCMV infection and to compete for DAG1 binding. In addition, we tested protamine sulfate (PS), a positively charged polycation and antagonist of heparin [34], for its ability to inhibit LCMV infection. To determine the binding specificity to heparin, we included chondroitin sulfate (CS) as another glycosaminoglycan (GAG) as a negative control. VSV WT or VSV-GP variants were pre-incubated with different concentrations of heparin or CS. In contrast, PS treatment was performed directly on cells before infection. Subsequently, cells were infected with VSV WT or VSV-GP and infection was quantified 15 h post infection (p.i.) by flow cytometry via eGFP expression.

**Fig 4:**
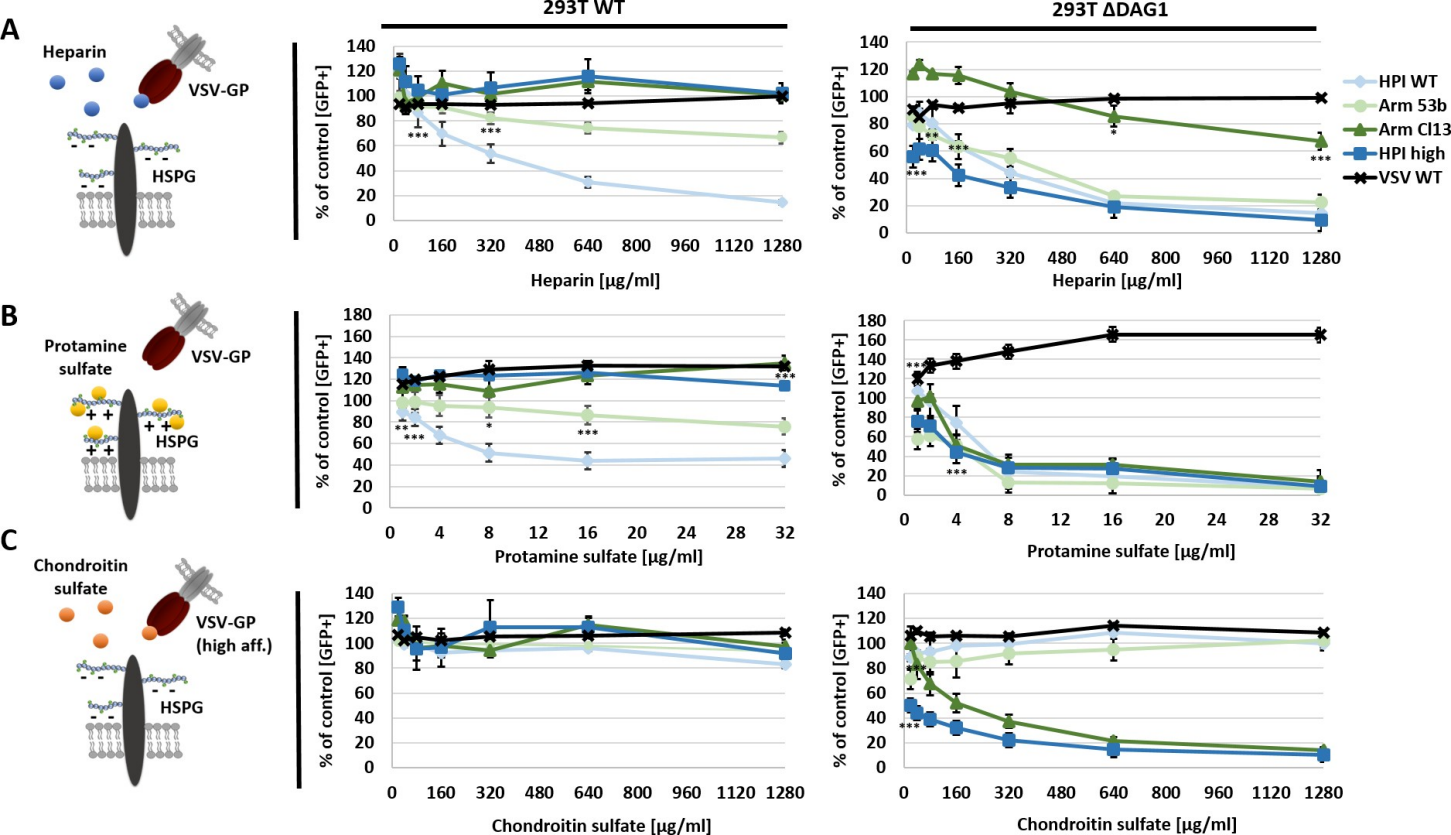
Treatment with soluble HS or PS efficiently inhibits infection of low affinity VSV-GP variants. VSV WT or VSV-GP variants (MOI 1) were incubated with 0, 20, 40, 80, 160, 320, 640 or 1,280 μg/ml of soluble (A) HS or (C) CS for 2 h at 4°C in a total volume of 50 μl PBS and afterwards used to infect 293T WT or Δ*DAG1* cells for 1 h at 37°C. To analyse the effect of (B) PS, cells were treated with 0, 1, 2, 4, 8, 16 or 32 μg/ml PS for 1 h at 37°C and subsequently infected with low or high affinity VSV-GP variants (MOI 1). Infection was quantified by flow cytometry measuring eGFP signal 15 h p.i. and is given relative to non-treated cells infected with the corresponding virus. Shown are the means ± SD of three replicates. Statistical analysis was performed using one-way ANOVA, followed by Turkey’s multiple comparison test; * p < 0.05, ** p < 0.001, *** p < 0.0001 (added below data points).

Infection with VSV WT could not be inhibited by treatment with soluble heparin, PS or CS in 293T WT or Δ*DAG1* cells (**Figs 4 and S3**). VSV-GP variants with low affinity LCMV-GP HPI WT (Y155H) or Arm 53b (L260F) were inhibited by soluble heparin or PS in a dose-dependent manner, while incubation with CS showed no effect. The inhibition was stronger for HPI WT (Y155H) compared to Arm 53b (L260F) in 293T WT cells, whereas both low affinity variants were efficiently inhibited by low concentrations of heparin or PS in Δ*DAG1* cells (**Figs 4 and S3**). The high affinity VSV-GP variants HPI high (H155Y) or Arm Cl13 (260L) showed no inhibition by HS, PS or CS in 293T WT cells. In Δ*DAG1* cells only HPI high (H155Y) was efficiently inhibited by soluble heparin. For all variants, strong inhibition at low concentrations of PS was observed in Δ*DAG1* cells. Surprisingly, only the two high affinity variants were prone to pre-incubation with soluble CS in Δ*DAG1* cells (**Figs 4 and S3**). Taken together, these results demonstrate heparin-related inhibition of low affinity variants in 293T WT cells and all variants in *ΔDAG1* cells.

### Chemical and genetic inhibition of sulfation inhibit VSV-GP infection

Next, we tested the role of sulfation, since one of the most enriched genes in the genome-wide CRISPR Cas9 knockout screen was *SLC35B2*, which encodes the Golgi-resident transporter of the universal sulfate donor 3’-phosphodenosine-5’-phosphosulfate (PAPS). Therefore, we cultured 293T WT and Δ*DAG1* cells for 2 weeks in the presence of 30 mM sodium chlorate, a specific inhibitor of PAPS synthetase [35]. Furthermore, we generated SLC35B2 knockouts for 293T WT (Δ*SLC35B2*) and DAG1 deficient (Δ*DAG1 SLC35B2*) cells. Infection of chemically treated or Δ*SLC35B2* cells was analysed with VSV WT or low and high affinity VSV-GP variants and quantified 15 h p.i. by flow cytometry via eGFP signal. Furthermore, 293T Δ*SLC35B2* and Δ*DAG1 SLC35B2* were included in the multi-step growth kinetic comparing high and low affinity VSV-GP variants (**S1 Fig**).

**Fig 5:**
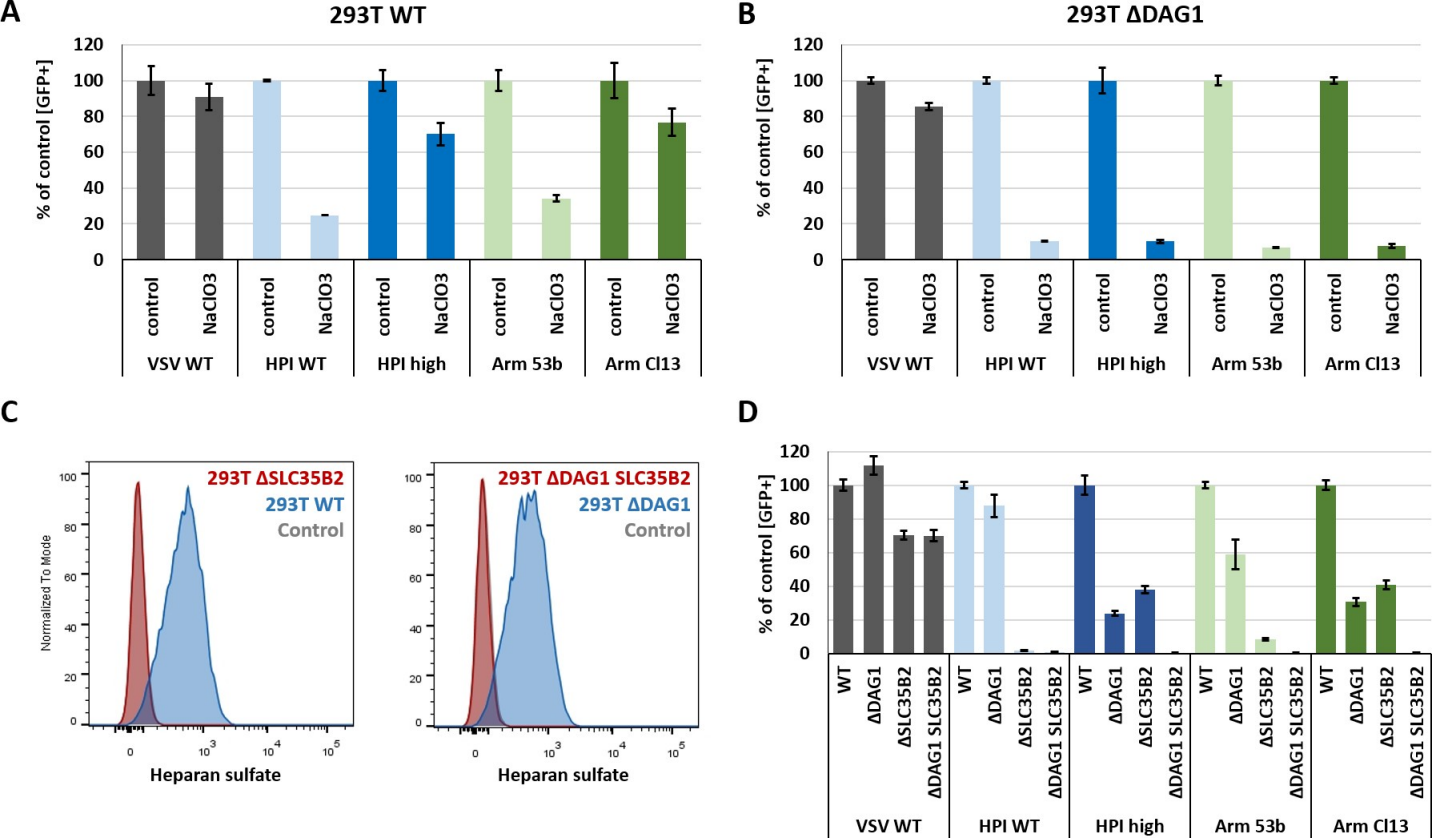
Chemical inhibition of sulfation and SLC35B2 knockout blocks infection of VSV-GP variants. For chemical inhibition, (A) 293T WT and (B) Δ*DAG1* cells were cultured for 2 weeks in the presence of 30 mM sodium chlorate (NaClO_3_) in complete growth medium. (C) Knockout of SLC35B2 in 293T WT (Δ*SLC35B2*) and DAG1 deficient cells (Δ*DAG1 SLC35B2*) was confirmed by heparan sulfate staining and flow cytometry analysis. Sodium chlorate treated and (D) knockout cells were infected at an MOI of 1 for 1 h at 37°C with VSV WT and low or high affinity VSV-GP variants. Infection was analysed by flow cytometry of eGFP signal 15 h p.i. Shown are the means ± SD of three replicates.

VSV WT infection was impaired by about 10–20% in 293T WT and Δ*DAG1* cells after treatment with sodium chlorate. In contrast, infection with low affinity variants HPI WT (Y155H) and Arm 53b (L260F) was reduced by about 70–80% and high affinity variants HPI high (H155Y) and Arm Cl13 (260L) by about 20–30% in 293T WT cells. Further reduction of about 90% was observed for all VSV-GP variants in sodium chlorate treated Δ*DAG1* cells (**Figs 5A and 5B and S2E**). Successful knockout of SLC35B2 was confirmed by HS staining and flow cytometry analysis (**Fig 5C**), as antibody detection (10E4) depends on N-sulfated residues within the epitope of HS chains. The results obtained for SLC35B2 knockout variants after infection with VSV WT or VSV-GP variants are stronger (**Fig 5D**) but in line with those generated for sodium chlorate treated 293T WT and Δ*DAG1* cells. These findings indicate that posttranslational sulfation of glycans or proteins in the Golgi compartment plays a major role for efficient infection of all tested VSV-GP variants, especially for low affinity variants and in the absence of functional DAG1.

### HSPG expression enables VSV-GP attachment but not entry in Burkitt lymphoma cells

**Fig 6:**
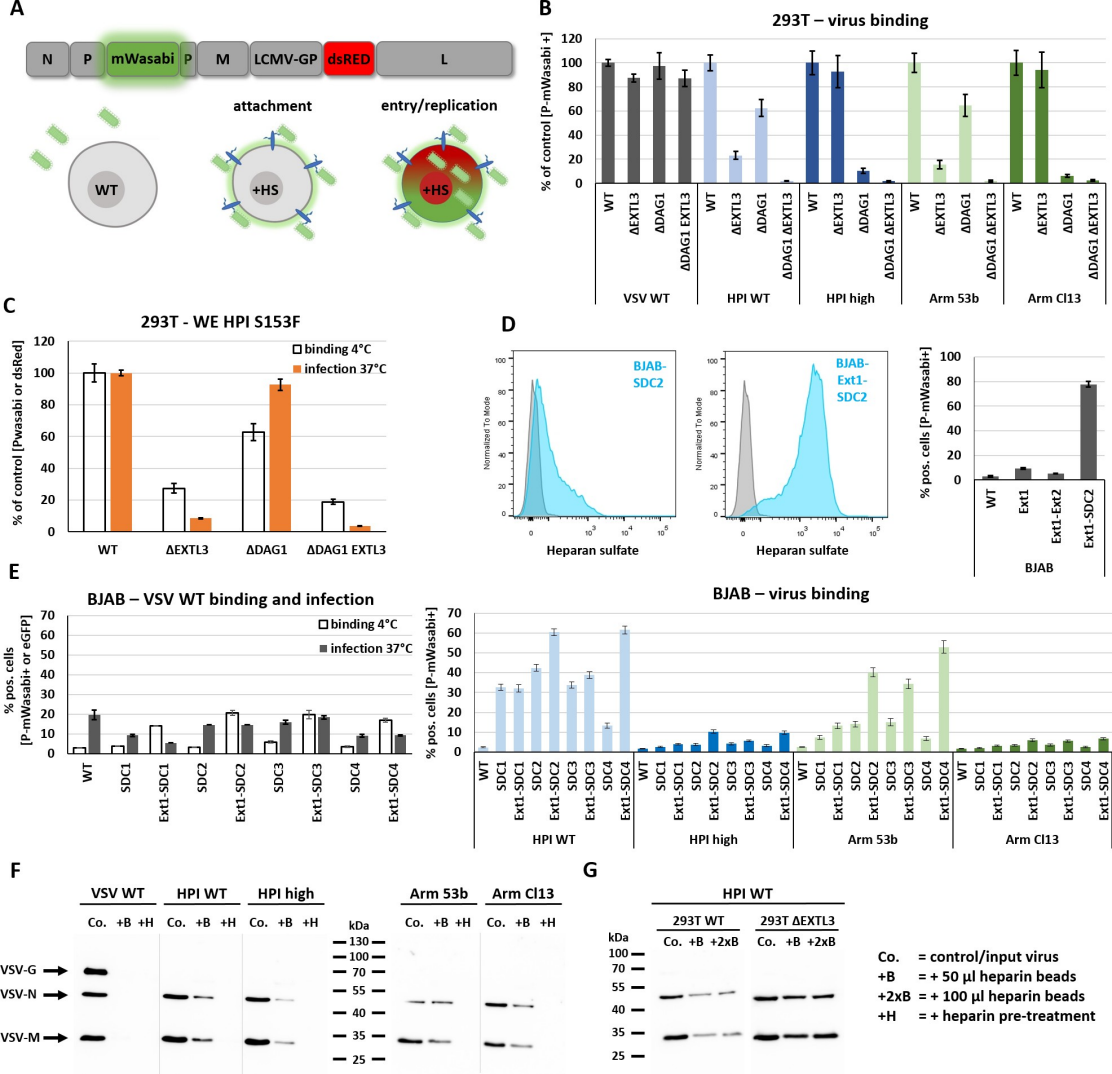
Proteoglycan expression restores HS expression on cell surface of BJAB cells and enables LCMV GP mediated virus attachment. (A) Schematic overview of a directly labelled VSV-GP construct encoding a P-mWasabi fusion construct and dsRed on 5^th^ position that allows to distinguish between attachment and virus entry/replication. Virus attachment was quantified by flow cytometry analysis. For attachment analysis, cells were incubated with directly labelled VSV-P-mWasabi-G or -GP variants for 30–45 min at 4°C in a total volume of 50 μl PBS or complete growth medium. Before measurement, samples were washed twice with PBS. Created with BioRender.com. (B) VSV-G and LCMV-GP mediated attachment of low or high affinity variants to 293T WT, Δ*EXTL3*, Δ*DAG1*, and Δ*DAG1 EXTL3* cells. (C) Binding (4°C for 30 min) and infection (MOI 1, 37°C, 14 h p.i.) assay with 293T WT and variants for WE HPI S153F carrying the low affinity mutation described for WE2.2. (D) Flow cytometry analysis of HS expression in BJAB cells stably transduced with SDC2 or the combination of EXT1-SDC2 (left). GP-mediated (HPI WT) virus attachment to BJAB cells stably transduced with EXT1, EXT1-EXT2 or EXT1-SDC2 (right). (E) Different members of the syndecan family (SDC1–4) were tested alone or in combination with EXT1 in BJAB cells for their ability to enable VSV-G or LCMV-GP mediated attachment and infection. (F) In a pulldown assay, 50 μl heparin-coated agarose beads (+B) were incubated with purified VSV WT or VSV-GP variants at 4°C for 4 h under constant rotation. After four washing steps with PBS + 0.2% BSA, the beads were resuspended in 100 μl RIPA buffer, incubated for 20 min on ice and boiled at 95°C for 10 min after the addition of 4x SDS loading buffer. Western blotting was performed with anti-VSV serum (detecting VSV-G, -N, -M). co. = same amount of input virus used for incubation with heparin-coated agarose beads. (G) Bead-based pulldown assay performed with purified VSV-GP HPI WT produced on 293T WT or 293T ΔEXTL3 cells. Signal intensity was compared using 50 μl or 100 μl heparin-coated agarose beads to control for bead saturation effects. A pre-stained page ruler was used with the range of 10–180 kDa. Shown are the means ± SD of three replicates.

HSPGs are often hijacked by viruses as attachment factors. In rare cases, these receptors are also used for virus entry [36]. Some members of the HSPG family, especially syndecans (SDC1–4), are known to function as autonomous endocytosis receptors [37]. This raises the question whether HSPGs could serve as alternative entry receptors for LCMV. To distinguish between attachment and entry/replication we designed a directly labelled VSV-reporter system, encoding a intramolecular fusion protein P-mWasabi [38] and a reporter dsRed. Virus binding can therefore be detected as a green (P-mWasabi) halo around cells via fluorescence microscopy or can be measured via flow cytometry. Virus uptake and replication results in a green and red signal within the cell due to the expression of P-mWasabi and dsRed (**Fig 6A**). The Burkitt lymphoma cell lines Raji and BJAB were used as a control, as they are completely resistant cells against LCMV infection. Interestingly, these cells lack HS on the cell surface, but exogenous expression of the HS elongation factors EXT1 or EXT2 can restore HS expression in several Burkitt lymphoma cell lines [39].

In 293T knockout variants, attachment of low affinity VSV-P-mWasabi-GP variants HPI WT (Y155H) or Arm 53b (L260F) in Δ*EXTL3* and Δ*DAG1* cells was reduced by about 80% and 40%, respectively. The high affinity VSV-P-mWasabi-GP variants HPI high (H155Y) or Arm Cl13 (260L) showed no altered attachment to Δ*EXTL3* cells, while a reduction of about 90% could be observed for Δ*DAG1* cells. Strongest reduction for all variants was observed in the double knockout cells (Δ*DAG1 EXTL3*). No major differences in virus attachment was observed for control VSV-P-mWasabi-G (**Fig 6B)**. Another low affinity variant (WE2.2) is characterized by a single point mutation at position S153F. To analyse and compare its dependence on HS expression for infection, we generated a directly labelled VSV-P-mWasabi-GP variant with the point mutation at S153F into the WE HPI high background. Both binding and infection studies in 293T WT and knockout clones revealed high dependence of HS expression, comparable to that of WE HPI WT variant (**Fig 6C**).

Surprisingly, in our experiments exogenous expression of EXT1, EXT2 or the combination of both did not restore cell surface HS expression in BJAB or Raji cells as described by Jarousse and colleagues (**S4B and S5A Figs**). In contrast, expression of core proteins like members of the syndecan or glypican family was sufficient to restore HS expression and to facilitate virus binding (**Fig 6D and 6E**). The co-expression of additional EXT1 further increased the HS signal (**Fig 6D**).

To address the question which HSPGs can serve as alternative entry receptors, we stably transduced BJAB and Raji cells with members of the syndecan (SDC1–4) or glypican family alone or in combination with EXT1 (**S4 and S5 Figs**). Attachment of VSV-P-mWasabi-G in BJAB or stable transduced BJAB cells with SDC1–4 was very low. Surprisingly, the combination of SDC1–4 with EXT1 increased VSV-P-mWasabi-G binding. This increase was not reflected in infection 12 h p.i. at 37°C (**Fig 6E**). As expected, low affinity VSV-P-mWasabi-GP variants attached more efficiently to cells expressing the HSPGs than high affinity variants. The data indicate a slight preference in virus binding to SDC2 and SDC4 when EXT1 is co-expressed (**Fig 6E**). Attachment could also be observed for members of the glypican family (not shown), indicating an unspecific role of HSPGs for virus attachment. Unfortunately, none of the tested HSPGs mediated virus entry in BJAB or Raji cells. Suppressed virus replication within BJAB or Raji cells can be excluded since the cells get readily infected with VSV WT. In addition, sporadic infections of SDC expressing BJAB and Raji cells with VSV-P-mWasabi-GP variants could be observed.

Next, we performed a pulldown assay by incubating purified VSV WT or VSV-GP variants with heparin-coated agarose beads. As an additional control, we pre-incubated VSV WT or VSV-GP variants with soluble heparin to block binding. Detection via western blot demonstrated no binding of VSV WT to heparin-coated agarose beads, similar as previously observed [30]. In contrast, pulldown of both low and high affinity VSV-GP variants was detected after incubation with heparin-coated agarose beads, while binding of all variants was sensitive to pre-incubation with 50 μg/ml soluble heparin (**Fig 6F**). The weaker signal of bound compared to input VSV-GP could be due to several reasons, such as naked virions, contaminating VSV proteins from virus stock production, saturation of binding sites on heparin-coated agarose beads, loss of beads during washing steps or host cell-derived HS that bound to the glycoprotein of LCMV on VSV-GP. To investigate some of these possibilities, we produced VSV-GP HPI WT on 293T WT or Δ*EXTL3* (lacking HS expression) cells and compared pulldown after using different amounts of heparin-coated agarose beads. Doubling the amount of beads had no major effect on virus pulldown, whereas production on Δ*EXTL3* cells strongly increased pulldown efficacy of VSV-GP HPI WT compared to virus produced on HS expressing 293T WT cells (**Fig 6G**).

In conclusion, these data demonstrate that all VSV-GP variants can bind to heparin and that low affinity VSV-GP variants efficiently use HSPG for virus attachment but not entry in Burkitt Lymphoma cell lines BJAB and Raji.

## Discussion

Differences in the binding affinity to the described cell receptor Dystroglycan-1 (DAG1) [2] of LCMV variants resulted in the classification of low and high affinity binders [12]. Single point mutations in the LCMV glycoprotein (GP) such as S153F, Y155H, and L260F [11–13] are responsible for the conversion of high into low affinity LCMV variants. Little or no differences in the infectivity of cells after DAG1 disruption suggest that low affinity variants can use an alternative receptor for cell entry. Several receptors such as DC-SIGN, LSECtin, Tyro3, and Axl have been published to serve as alternative DAG1-independent receptors [15]. However, the role of Axl remains controversial, as no effect on LCMV infection was observed in an Axl knockout mouse model [16]. Furthermore, the susceptibility of 293T Δ*DAG1* cells cannot be explained by the use of the aforementioned surface receptors, as they are not or weakly expressed by 293T cells. Therefore, the aim of this study was to identify host factors involved in the uptake of low affinity LCMV variants in the absence of functional DAG1. To analyse this, we performed a genome-wide CRISPR Cas9 knockout screen in 293T Δ*DAG1* cells. For cell selection, we used a vesicular stomatitis virus (VSV)-based reporter system, equipped with the glycoprotein (GP) of different LCMV GP variants and *eGFP* as a marker gene at the 5^th^ position of the virus genome.

Several attempts to perform selection for cells resistant to VSV-GP infection with replication competent virus (high or low affinity LCMV GP variants) were not successful, indicating that VSV-GP, in the absence of DAG1, could either use several alternative receptors, a family of receptors with high similarity, or a non-specific entry pathway like apoptotic mimicry. On cellular basis, this observation may also indicate an essential role for the alternative receptor in cell survival, cell adhesion or cell growth, thereby preventing selection of resistant knockouts. The use of a gentler approach by infection with replication incompetent virus allowed successful isolation of less susceptible cells. Deep sequencing of this cell population revealed an important role of HS biosynthesis in low affinity LCMV infection. HS has already been identified as an important entry factor for other viruses such as HERV-K, RVFV, ZIKV, MVA, or SINV [26–30]. Interestingly, similar to our findings, HSPGs also appear to play an important role in DAG1-independent entry of Lassa Virus (LASV) [17,40]. The authors performed a haploid genetic screen using cells lacking α-DAG1 and recombinant VSV, pseudotyped with the GP of LASV. They demonstrated the importance of LAMP1 for LASV infection, but also observed an increase of enriched factors involved in heparan sulfate biosynthesis (e.g. EXT1, EXTL3, SLC35B2, B3GAT3, EXT2 etc.) [40], similar to our results. We were able to prove the critical role of HS biosynthesis by single and double knockouts of *DAG1* and/or *EXTL3*, an essential factor in the elongation process of HS chains [32].

Treatment with sodium chlorate, a specific inhibitor of the universal sulfate donor 3’-phosphoadenosine-5’-phosphosulfate (PAPS) synthesis [35], and the knockout of SLC35B2, a Golgi-resident transporter of PAPS, demonstrated the importance of sulfation for efficient infection with high and low affinity variants. PAPS serves as an important substrate for sulfation of several proteins as CCR5 [41] and all members of glycosaminoglycans (GAGs) such as chondroitin [42] or heparan sulfate [43] in the Golgi compartment. It is therefore not surprising that SLC35B2 frequently appears in genome-wide screens as a top hit [25,30,41,44]. Thus, it cannot be excluded that other important host factors for LCMV infection are also affected, as might be indicated by reduced infection of high affinity VSV-GP variants in 293T Δ*SLC35B2* cells.

In addition to the genetic and chemical approach, we could also confirm the importance of cell surface HSPG by enzymatic digestion of HS chains using Heparinase I/III. Although the reduction of HS on the cell surface was not complete, it was sufficient to achieve a similar decrease of infection as observed for knockout of *EXTL3*.

In contrast to our observations, LCMV was found not to interact with HS in previous studies [45–47]. However, these studies mainly analysed the high affinity variant Arm Cl13, which could not or weakly be inhibited in our study as well. Furthermore, no study analysed the importance of HS in the absence of functional DAG1. Strong inhibition of binding observed in Δ*EXTL3* cells for Arm 53b (L260F), HPI WT (Y155H) and WE HPI S153F (S153F) indicate an important role of these mutations for GP-mediated attachment. Indeed, the switch from DAG1 to HS binding is plausible, because DAG1 and heparin share an overlapping binding region within the C-terminally located G-like domain 4 (LG4) of the laminin α1 chain, as previously discussed by Rojek and colleagues [45]. Consequently, this leads to competition between heparin and DAG1 for laminin α1 binding [48]. Competition can also be observed between LCMV GP and laminin α1 because they share overlapping binding regions within DAG1 [46]. In this context it is also interesting to mention that the HSPGs agrin and perlecan harbour LG domains that enable direct binding to DAG1 as well [5].

An unexpected observation in our study was the strong inhibition with either chondroitin sulfate (CS) or both CS and protamine sulfate (PS) for the high affinity variants HPI high or Arm Cl13, respectively. Usually CS is used as a negative control to prove specific binding to HS, which could be shown for the low affinity variants HPI WT and Arm 53b. The recent findings that PS also functions as a CS inhibitor [49] and low affinity binding of CS to laminin [50], could indicate a role of CSPGs as alternative receptors for high affinity variants in the absence of the preferred receptor DAG1.

To address the question whether HSPGs are used for virus attachment or virus entry, we designed a directly labelled VSV-GP construct by intramolecular insertion of the green fluorescent protein mWasabi with the P protein of VSV. At the same time a second reporter gene dsRed, which is encoded at the 5^th^ position of the VSV genome, allows to distinguish between virus attachment and virus entry/replication. We decided to use the Burkitt lymphoma cell lines Raji and BJAB, because, unlike most other lymphocyte cell lines, they are completely resistant to LCMV infection and do not express HS [39]. In contrast to Jarousse and colleagues, expression of proteoglycan members, e.g. from the syndecan (SDC) or glypican (GPC) family was sufficient to restore HS expression on the cell surface. We decided to focus on the transmembrane HSPG members SDC1–4. As widely expressed surface receptors with a C-terminal cytoplasmic domain, SDCs are involved in cell signalling and biological functions like cell migration, adhesion and cell-cell contact. They can interact with other cell surface receptors like integrins and growth factor receptors or mediate binding to extracellular matrix proteins such as collagen, laminin or fibronectin [51]. For most HS binding viruses, SDCs are described to serve as attachment and not entry receptors with one exception: 6-O- and N-sulfated SDC1, which serves as an attachment and entry receptor for baculovirus [36]. In general, HSPGs as autonomous endocytosis receptors have been known for some time [37] and could therefore be considered as alternative entry receptors for LCMV. We could show a clear binding of low affinity LCMV variants to SDC1–4, which in most cases could be increased by additional EXT1 expression. This finding can be explained by increased HS biosynthesis as shown by a stronger HS signal in flow cytometry analysis. Other HSPGs, such as GPC1, could also be used by low affinity LCMV variants, indicating an unspecific role of HSPGs for virus attachment. Differences in virus binding between SDC members might result from their non-conserved ectodomain. Besides differences in the number and positioning of HS chains, they can also contain additional CS chains (SDC1 and 3). In these terms, SDC2 and SDC4 share more similarities, which would explain the preference of both receptors. None of the tested HSPGs mediated virus uptake, indicating that these receptors in BJAB and Raji cells serve only as attachment factors. Suppressed viral replication can be ruled out because VSV WT efficiently infects and replicates in these cells. In addition, we observed sporadic and most likely random VSV-GP uptake and virus replication in SDC expressing BJAB and Raji cells. Since a lack of virus uptake may also depend on the absence of cell-specific signalling factors or the ability to perform certain endocytosis pathways, these observations are only valid for BJAB and Raji cells.

Specific binding of VSV-GP to heparin was demonstrated by a pulldown assay using heparin-coated agarose beads. Binding of both, low and high affinity VSV-GP variants was detected, indicating that all tested variants are able to interact with heparin. This result is consistent with our data showing that high affinity variants bind to HSPG in the absence of functional DAG1. Increased pulldown of VSV-GP HPI WT produced on 293T Δ*EXTL3*, lacking HS biosynthesis, compared with virus produced on 293T WT cells suggests, that GP of viral progeny may be covered with heparin.

The results from this study (for HSPG) and an earlier publication by Kunz et al. (2003, for DAG1) suggest that HSPG and DAG1 [52] serve as attachment factors for low and high affinity variants, respectively. As residual virus infection is seen in knockout cells for both receptors and for both high and low affinity viruses, both LCMV variants might use the same, still unknown entry receptor (family) for membrane fusion and virus uptake.

## Materials and methods

### Cell lines and plasmids

293T (ATCC, Manassas, VA, USA), L929 (ACC 2, DSMZ, Braunschweig, Germany) and derivates were cultured in high glucose Dulbecco’s Modified Eagle Medium (Merck, Darmstadt, Germany) supplemented with 10% fetal bovine serum (FBS, Gibco, Carlsbad, CA, USA), 2% L-Glutamin (200 mM, Gibco), 100 U/ml Penicillin-Streptomycin (10,000 U/ml, Gibco), 1% MEM Non-Essential Amino Acids Solution (100x, Gibco), and 1% Sodium Pyruvate (100 mM, Gibco). BJAB (ACC757, DSMZ), Raji (CCL-86, ATCC) and derivates were cultured in RPMI-1640 medium (Gibco) supplemented with 10% FBS, 2% L-Glutamin and 100 U/ml Penicillin-Streptomycin. BHK-21 (ATCC) were maintained in Glasgow minimum essential medium (Gibco) supplemented with 10% FBS, 5% tryptose phosphate broth (Gibco), and 100 U/ml Penicillin-Streptomycin. All cell lines were cultured in humidified incubators at 37°C and 5% CO_2_.

Human *EXT1*–*2*, *SDC1*–*4* and *GPC1* sequences were amplified from 293T cDNA and subcloned into a lentiviral vector (#119863; pLenti CMVie-IRES-BlastR; Addgene, Watertown, USA). Additional selection variants were cloned by exchanging the blasticidin resistance with that of puromycin (#1000000049; Addgene) or hygromycin (Hygromycin plasmid; Genecopoeia, Rockville, MD, USA). All PCR primers are listed in detail in S2 Table. Lentiviruses were produced as described below. 293T, BJAB and Raji cells were transduced using spinfection in a 12-well format at 1,000 g for 2 h at 32°C in the presence of 8 μg/ml Polybrene (Sigma-Aldrich, St. Louis, MO, USA). Clonal isolation of Cas9-2A-CD8 expressing 293T ΔDAG1 cells was performed by sorting CD8 positive cells via flow cytometry. Cas9 expression was verified by western blot analysis (7A9-3A3 antibody; Santa Cruz Biotechnology, Dallas, TX, USA). BJAB, Raji or 293T cells stably transduced with vectors encoding for resistance markers were selected with either 500 μg/ml hygromycin (Invivogen, Toulouse, FR), 12.5 μg/ml blasticidin (Invivogen) or 0.5 μg/ml puromycin (Invivogen), respectively.

### Virus variants

VSV, VSV-ΔG-GP (pseudotyped, single round infectious) and VSV-GP (chimera of VSV and LCMV GP) have been described previously [23,53]. Five different LCMV GP variants WE HPI S153F (WE2.2), WE HPI (Y155H), WE high (H155Y), Arm 53b (L260F), and Arm Cl13 (260L) were compared in this study (**Fig 1A and S1 Table**). Reporter VSV-GP variants contain additional eGFP at position 5 of the viral genome. Directly labelled VSV-PmWasabi-GP variants were generated by exchanging P with an intramolecular fusion protein P-mWasabi [38] and cloning dsRed at position 5 (**Fig 6A**). All VSV-GP variants were rescued using a helper virus-free rescue protocol as described previously [54]. Briefly, viruses were rescued in 293T WT cells by co-transfection of helper plasmids using the calcium phosphate method and plaque purified on BHK-21 cells. Working stocks were produced on BHK-21 cells, filtered (0.45 μM), concentrated overnight by low speed centrifugation using a 20% sucrose cushion, aliquoted and stored at—80°C. Virus variants were titrated via tissue culture infectious dose 50% (TCID50) on BHK-21 or 293T cells as described below. VSV-ΔG-GP were produced and titrated on BHK-21 cells stably expressing LCMV GP.

LCMV working stocks of the strains Arm Cl13, Arm 53b (both kindly provided by Annette Oxenius, Prof.), and WE HPI were produced in a biosafety 3 lab on BHK-21 cells. Briefly, 80% confluent BHK-21 cells were infected with an MOI of 0.01 at 37°C in ¼ of the total volume of GMEM supplemented with 2% FBS. One hour later, the remaining amount of GMEM was added. The virus containing supernatant was collected and pooled 48 h and 72 h post infection. After centrifugation at 1,000 g for 5 min at 4°C, aliquots were stored at—80°C. LCMV titers were determined as previously described [55]. Briefly, BHK-21 cells were infected in serial dilutions and LCMV-NP (VL-4; **S4 Table**) positive cells were measured 24 h later via flow cytometry.

To produce lentiviral particles, we adapted a previously described method [56]. Briefly, 10 cm dishes with 3.3x10^6^ 293T cells seeded the previous day were co-transfected using the calcium phosphate method with 12.5 μg lentiviral Gag/Pol plasmid pCMV-dR8.91, 1 μg of VSV-G or LCMV-GP plasmid and 7.5 μg lentiviral transfer vector plasmid (encoding for eGFP, Cas9-2A-CD8, SDC1–4, GPC1, EXT1–2 or GeCKO Library A/B) in the presence of 25 μm chloroquine. The medium was exchanged 14 h post transfection. The supernatant was harvested and pooled after 36 and 60 h. Following filtration (0.45 μm filter), lentivirus was either further purified and concentrated by low speed centrifugation using a 20% sucrose cushion or aliquoted directly and stored at—80°C. Virus titers were determined by limited dilution and flow cytometry analysis of transduced cells (eGFP+) or by the colorimetic MTT assay analysing resistant cells after stable transduction with vectors encoding resistance marker.

### TCID50 assay

Viral titers were determined and calculated via TCID50 assay according to the Spearman-Kaerber formula [57]. Briefly, the day before infection, 10^3^ BHK-21 cells were seeded per well of a 96-well plate. Eight replicates of ten-fold serial dilutions of virus samples were added to the cells and incubated at 37°C. One week after infection, the wells with a clear cytopathic effect (CPE) were counted and the titer was calculated.

### Generation of knockout cells

293T and L929 cells were transfected with a CRISPR-Cas9-2A-eGFP plasmid (#PX458, Addgene) encoding the indicated gRNA (**S3 Table**). The gRNAs were designed using the ATUM gRNA Design Tool. Clonal lines were generated by limited dilution and verified by flow cytometry and sequencing of the genomic target region (Microsynth, Balgach, Switzerland).

### Genome-wide CRISPR Cas9 knockout screen

Three rounds of independent selection experiments of pooled genome-wide CRISPR Cas9 knockout screens were performed using the human GeCKOv2 CRISPR knockout pooled library (a gift from Feng Zhang; # 1000000049; Addgene) [58]. The screen was performed according to a protocol adapted from a previous study [31]. For each replicate 3x10^8^ 293T ΔDAG1 cells stably expressing Cas9-2A-CD8 were transduced with the lentiviral packed GeCKO library at an MOI of 0.3 in the presence of 8 μg/ml Polybrene (Sigma-Aldrich). Twenty-four hours later the cells were selected for 10 days with 0.8 μg/ml puromycin. Subsequently, 3x10^8^ cells were challenged with replication competent VSV-GP variants (MOI 1) or single round infectious VSV-ΔG-GP WE HPI (MOI 10). During virus mediated selection, uninfected cells were cultured as controls. After three rounds of infection, genomic DNA of 5x10^6^ selected or 3x10^7^ uninfected cells was isolated using the Monarch Genomic DNA Purification Kit (NEB). In a nested PCR approach using Herculase II Fusion DNA Polymerase (Agilent, Santa Clara, CA, USA), we first pre-amplified the sgRNA containing lentiviral insertions and in the second round added stagger bases and illumina P5- and P7-barcoded adaptors (**S5 Table**). The PCR products were then analysed by agarose-gel electrophoresis (AGE) and pooled in equal concentrations. Subsequently, the pooled PCR amplicons were gel purified and deep sequenced on an Illumina HiSeq4000 in collaboration with the biomedical sequencing facility (BSF) of the Research Center for Molecular Medicine of the Austrian Academy of Sciences (CeMM). Sequencing data were analysed with the online tools GenePattern [59] and Galaxy [60]. Reads were then demultiplexed and trimmed to align sgRNA sequences to a reference using Bowtie2 [61]. Sequencing data are available in the supplements (**S6 Table**).

### Flow cytometry analysis

Cells were detached using 5 mM EDTA and washed twice with FACS Buffer (PBS with 1% FBS). We stained with the indicated antibodies (**S4 Table**) at 4°C for 30–60 min followed by two washing steps with FACS Buffer. For intracellular staining (LCMV N), the cells were treated with the fixation/permeabilization kit (Transcription Factor Buffer Set; BD Biosciences, San Jose, CA, USA) according to the manufacturers protocol. Each measurement included 1x10^4^ events and was performed on a FACSCanto II (BD Biosciences) and analysed using FlowJo software.

### Heparinase I/III and sodium chlorate treatment

293T and L929 cells were treated in complete growth medium with 2 Units/ml Heparinase I/III (Sigma-Aldrich) for 2 h at 37°C. Cleavage and reduction of cell surface heparan sulfate (HS) was verified by flow cytometry analysis.

293T and L929 cells were passaged for 2 weeks in complete growth medium supplemented with 30 or 50 mM sodium chlorate (Roth).

Post-treatment, supernatant was removed and cells were infected with VSV WT or VSV-GP variants for 1 h at 37°C in complete growth medium at an MOI of 1. Infection was assessed 15 h p.i. by flow cytometry (eGFP) and normalized to untreated controls.

### Inhibition assay

Heparin, Chondroitin sulfate (CS) and protamine sulfate (PS; Carl Roth, Karlsruhe, Germany) were dissolved in PBS. High and low affinity VSV-GP variants (MOI 1) were pre-incubated with the indicated amounts of Heparin or CS (for both: 20, 40, 80, 160, 320, 640 or 1,280 μg/ml) for 2 h at 4°C in a total volume of 50 μl. Treatment with PS was performed directly on cells with the indicated concentrations (1, 2, 4, 8, 16, 32 μg/ml) for 1 h at 37°C. Subsequently, cells were infected with VSV WT or VSV-GP variants for 1 h at 37°C. Infection was assessed 15 h p.i. by flow cytometry (eGFP) and normalized to untreated controls.

### Binding and heparin pulldown assay

Directly labelled VSV-P-mWasabi-G or -GP variants by intramolecular fusion protein of VSV-P and the green fluorescent protein mWasabi (P-mWasabi) [38] were designed and produced as mentioned earlier. Working stocks were titrated via TCID50 on 293T WT cells. For all experiments 1x10^5^ cells were incubated in 50 μl PBS or complete growth medium at 4°C with an MOI of 10 (corresponds to approximately 50% virus-bound 293T WT cells). After 30–45 min, cells were washed twice with PBS and binding was quantified by flow cytometry (% of mWasabi positive cells). Virus replication could be detected by intracellular expression of P-mWasabi and dsRed as early as 12 h after infection.

For the pulldown assay, the inner surface of 1.5 ml screw cap tubes were blocked over night with PBS containing 5% bovine serum albumin (BSA, Roth) at 4°C under constant rotation. VSV WT or VSV-GP variants were pre-treated with or without 50 μg/ml soluble Heparin in 100 μl PBS + 0.2% BSA for 2 h at 4°C. Fifty or 100 μl of heparin-coated agarose beads (Sigma-Aldrich) were washed three times and incubated with pre-treated VSV WT or VSV-GP variants for 4 h at 4°C under constant rotation. Subsequently, samples were washed four times with PBS + 0.2% BSA, resuspended in 100 μl RIPA lysis buffer, incubated on ice for 20 min and boiled at 95°C for 10 min after addition of 4x SDS loading buffer. Samples were loaded on a 10% polyacrylamide gel and transferred to a nitrocellulose membrane. After blocking at room temperature for 1 h with PBS containing 5% skim milk and 0.1% Tween 20 (PBSTM), the membrane was incubated over night at 4°C with anti-VSV serum (1:40,000 in PBSTM, Prof. Stefan Finke, Friedrich Loeffler Institute, Island Riems), followed by peroxidase-conjugated goat anti-rabbit IgG (1:10,000 in PBST, Jackson ImmunoResearch) for 1 h at room temperature. Blots were developed with enhanced chemiluminescence (ECL).

### Statistical analysis

Statistical analysis was performed using GraphPad prism software (version 9, GraphPad Software, La Jolla, CA, USA), as indicated in the figure legends.

## Supporting information

S1 Fig Multi-step growth kinetic of VSV WT or VSV-GP variants in 293T knockout cells. 1x10^5^ cells (293T WT, Δ*DAG1*, Δ*EXTL3*, Δ*SLC35B2*, Δ*DAG1 EXTL3*, Δ*DAG1 SLC35B2*) were seeded per well in a 24 well plate. One day later, cells were infected with an MOI of 0.01 with VSV WT or high and low affinity VSV-GP variants. The supernatant was collected 8, 16, 24, 36 and 48 h p.i. and stored at—80°C. Virus titre was determined by TCID50 on BHK-21 cells. Shown are the means ± SD of two replicates.
(TIF)

S2 Fig HS expression is important for the infection of DAG1 deficient L929 cells. (A) Flow cytometry analysis of Heparan sulfate (10E4) expression in L929 WT (red) and two clones of Δ*EXTL3* L929 cells (blue) and (C) after treatment with 1 Unit Heparinase I/III for 2 h at 37°C. Infection assays comparing the susceptibility of L929 WT cells vs (B) L929 Δ*EXTL3*, (D) Heparinase I/III treated L929 WT cells and (E) L929 WT cells cultured for 2 weeks in the presence of 50 mM sodium chlorate (NaClO_3_). Cells were infected with VSV WT (control, grey) and high or low affinity VSV-GP variants at an MOI of 5 for 1 h at 37°C. Infected cells were quantified 15 h later via measurement of eGFP positive cells by flow cytometry. Shown are the means ± SD of three replicates.
(TIF)

S3 Fig Treatment with soluble HS or PS inhibits infection of low affinity VSV-GP variants in L929 WT cells. VSV WT (control) and high or low affinity VSV-GP variants were pre-incubated with different amounts of soluble (A) HS or (B) CS for 2 h at 4°C. Afterwards L929 WT cells were infected for 1 h at 37°C. (C) Inhibitory effect of PS. L929 WT cells were pre-treated with different concentrations of PS for 1 h at 37°C and subsequently infected with VSV WT (control) and high or low affinity VSV-GP variants. Infection was quantified 15 h p.i. by flow cytometry measuring eGFP expression. Shown are the means ± SD of three replicates.
(TIF)

S4 Fig SDC and HS staining of 293T WT and transduced BJAB cells. Flow cytometry analysis of (A) SDC1–4 expression (blue) in 293T WT cells. HS expression analysis via Flow cytometry of BJAB cells stably transduced with lentiviral vectors encoding for (B) EXT1–2 and (E) EXT1 + SDC1–4 stably transduced BJAB cells. SDC expression analysis of BJAB cells stably transduced with (C) SDC1–4 or (D) in combination with EXT1. Cells were selected 48 h post transduction either with blasticidin (SDC vectors) or puromycin (EXT vectors). Staining controls, single transduced BJAB cells (EXT1) or WT BJAB cells are marked in grey.
(TIF)

S5 Fig SDC and HS staining of Raji cells. Flow cytometry analysis HS expression in Raji cells (A) stably expressing EXT1-EXT2 and (B) EXT1 + SDC1–4 stably transduced cells. (C) SDC staining of Raji-EXT1 cells stably transduced with SDC1–4. Cells were selected 48 h post transduction either with blasticidin (SDC vectors) or puromycin (EXT vectors). Marked in grey are either single transduced Raji cells (EXT1) or the WT.
(TIF)

S1 Table Comparison of high and low affinity LCMV variants. (DOCX)

S2 Table Human and murine CRISPR gRNAs. (DOCX)

S3 Table Cloning primer for lentiviral transfer vector. Italic sequence: overhang.
(DOCX)

S4 Table Antibodies for Flow cytometry analysis. (DOCX)

S5 Table NGS primer for nested PCR. (DOCX)

S6 Table NGS data on genome-wide CRISPR Cas9 knockout screen performed with VSV-ΔG-GP HPI WT in 293T Δ*DAG1* cells. (XLSB)
