# Supplementary figures and images for: Heparan sulfate proteoglycans serve as alternative receptors for low affinity LCMV variants
Source: PLoS Pathog. 2021 Oct 14;17(10):e1009996. doi: 10.1371/journal.ppat.1009996 (PMC8547738; doi:10.1371/journal.ppat.1009996)

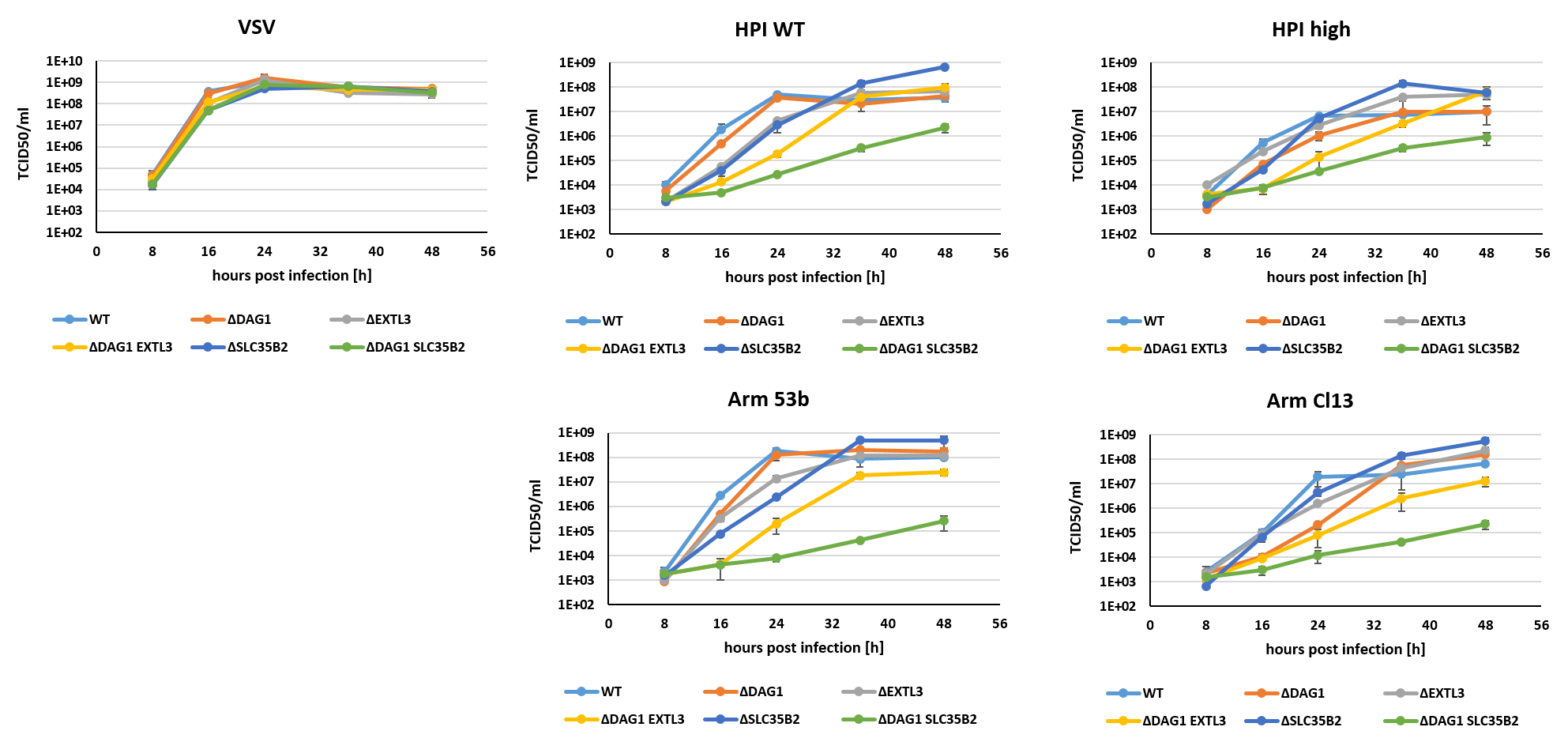

Supplement: S1 Fig — 1x10^5^ cells (293T WT, ΔDAG1, ΔEXTL3, ΔSLC35B2, ΔDAG1 EXTL3, ΔDAG1 SLC35B2) were seeded per well in a 24 well plate. One day later, cells were infected with an MOI of 0.01 with VSV WT or high and low affinity VSV-GP variants. The supernatant was collected 8, 16, 24, 36 and 48 h p.i. and stored at—80°C. Virus titre was determined by TCID50 on BHK-21 cells. Shown are the means ± SD of two replicates. (TIF) [file ppat.1009996.s001.tif]

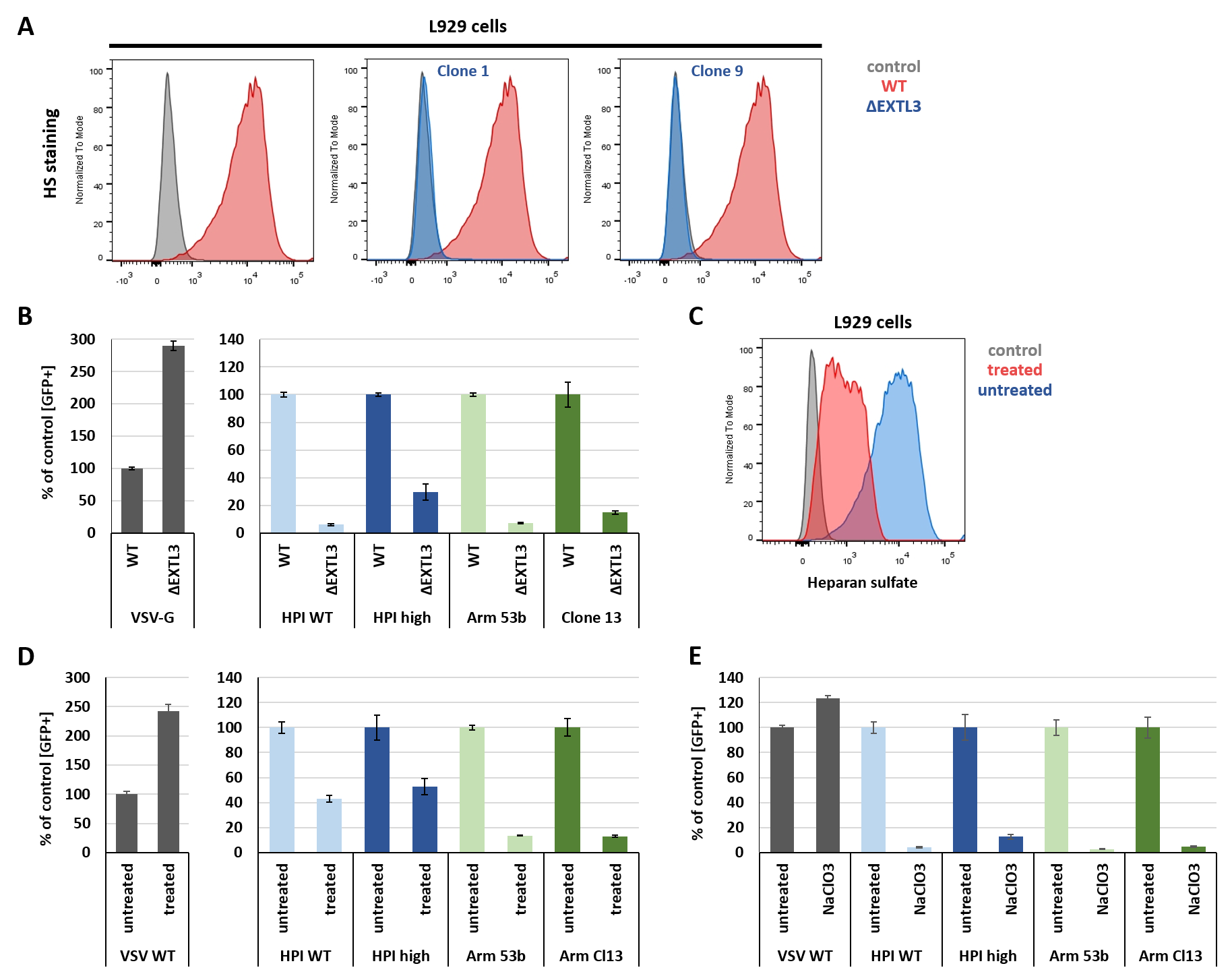

Supplement: S2 Fig — (A) Flow cytometry analysis of Heparan sulfate (10E4) expression in L929 WT (red) and two clones of ΔEXTL3 L929 cells (blue) and (C) after treatment with 1 Unit Heparinase I/III for 2 h at 37°C. Infection assays comparing the susceptibility of L929 WT cells vs (B) L929 ΔEXTL3, (D) Heparinase I/III treated L929 WT cells and (E) L929 WT cells cultured for 2 weeks in the presence of 50 mM sodium chlorate (NaClO3). Cells were infected with VSV WT (control, grey) and high or low affinity VSV-GP variants at an MOI of 5 for 1 h at 37°C. Infected cells were quantified 15 h later via measurement of eGFP positive cells by flow cytometry. Shown are the means ± SD of three replicates. (TIF) [file ppat.1009996.s002.tif]

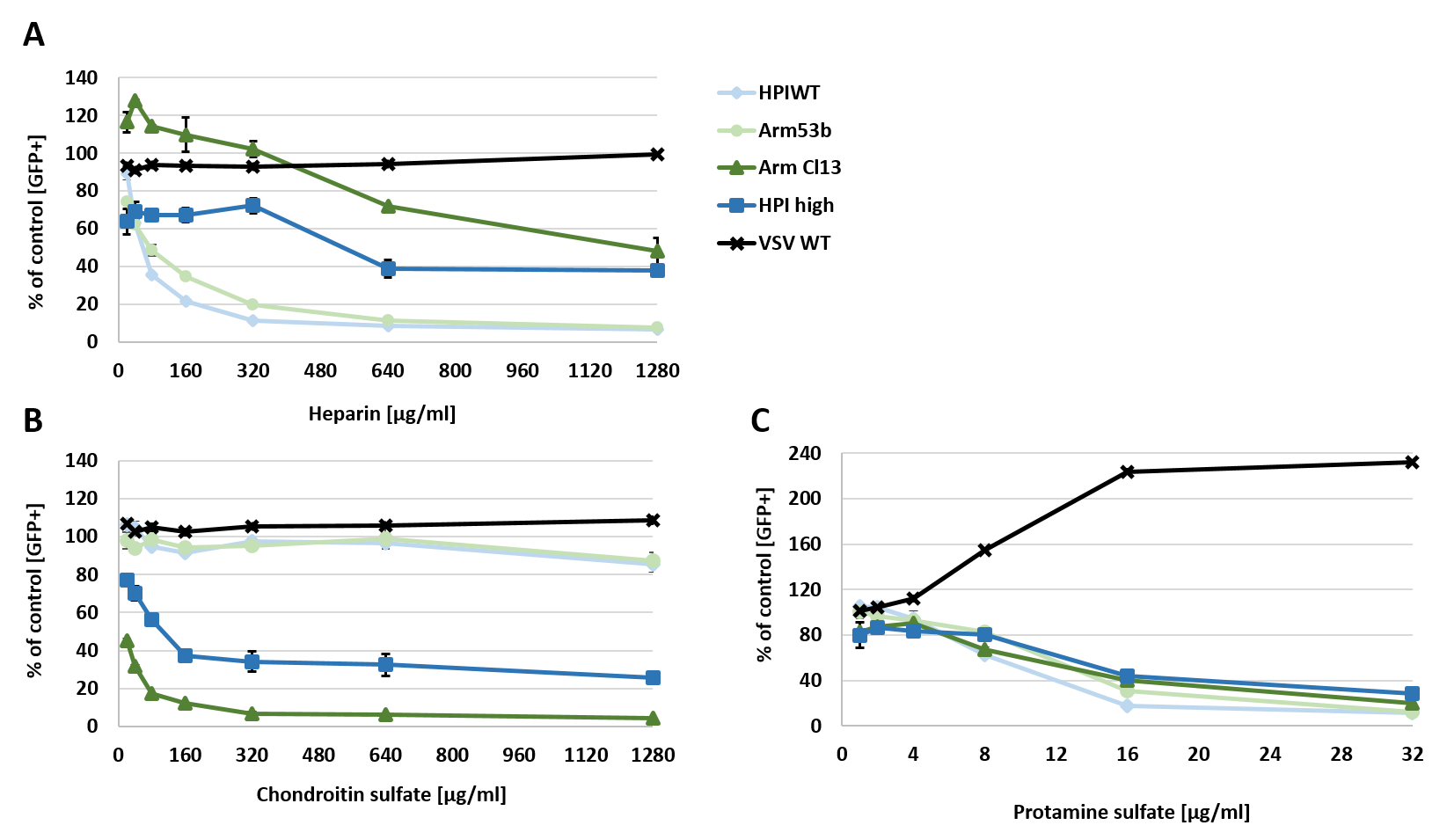

Supplement: S3 Fig — VSV WT (control) and high or low affinity VSV-GP variants were pre-incubated with different amounts of soluble (A) HS or (B) CS for 2 h at 4°C. Afterwards L929 WT cells were infected for 1 h at 37°C. (C) Inhibitory effect of PS. L929 WT cells were pre-treated with different concentrations of PS for 1 h at 37°C and subsequently infected with VSV WT (control) and high or low affinity VSV-GP variants. Infection was quantified 15 h p.i. by flow cytometry measuring eGFP expression. Shown are the means ± SD of three replicates. (TIF) [file ppat.1009996.s003.tif]

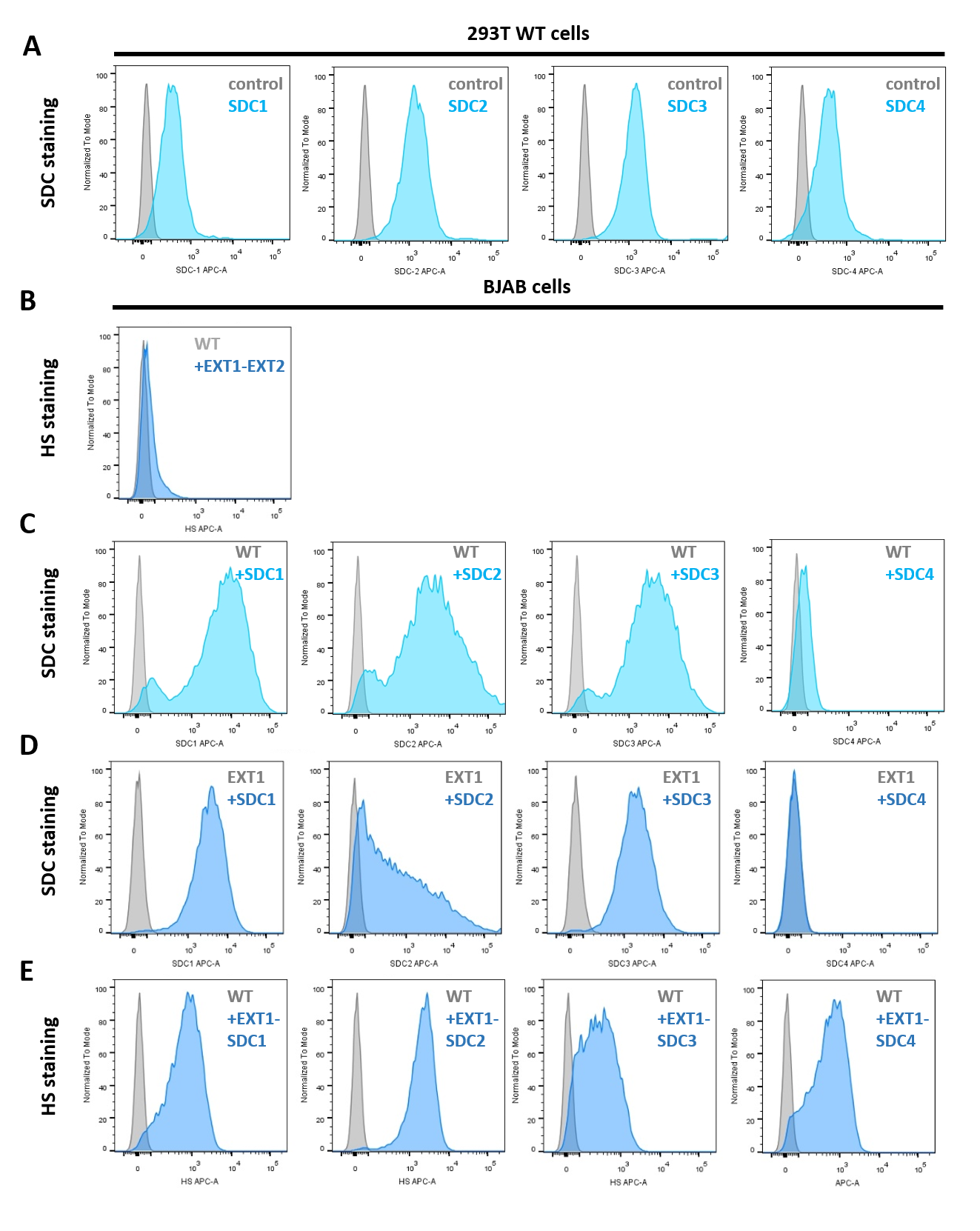

Supplement: S4 Fig — Flow cytometry analysis of (A) SDC1–4 expression (blue) in 293T WT cells. HS expression analysis via Flow cytometry of BJAB cells stably transduced with lentiviral vectors encoding for (B) EXT1–2 and (E) EXT1 + SDC1–4 stably transduced BJAB cells. SDC expression analysis of BJAB cells stably transduced with (C) SDC1–4 or (D) in combination with EXT1. Cells were selected 48 h post transduction either with blasticidin (SDC vectors) or puromycin (EXT vectors). Staining controls, single transduced BJAB cells (EXT1) or WT BJAB cells are marked in grey. (TIF) [file ppat.1009996.s004.tif]

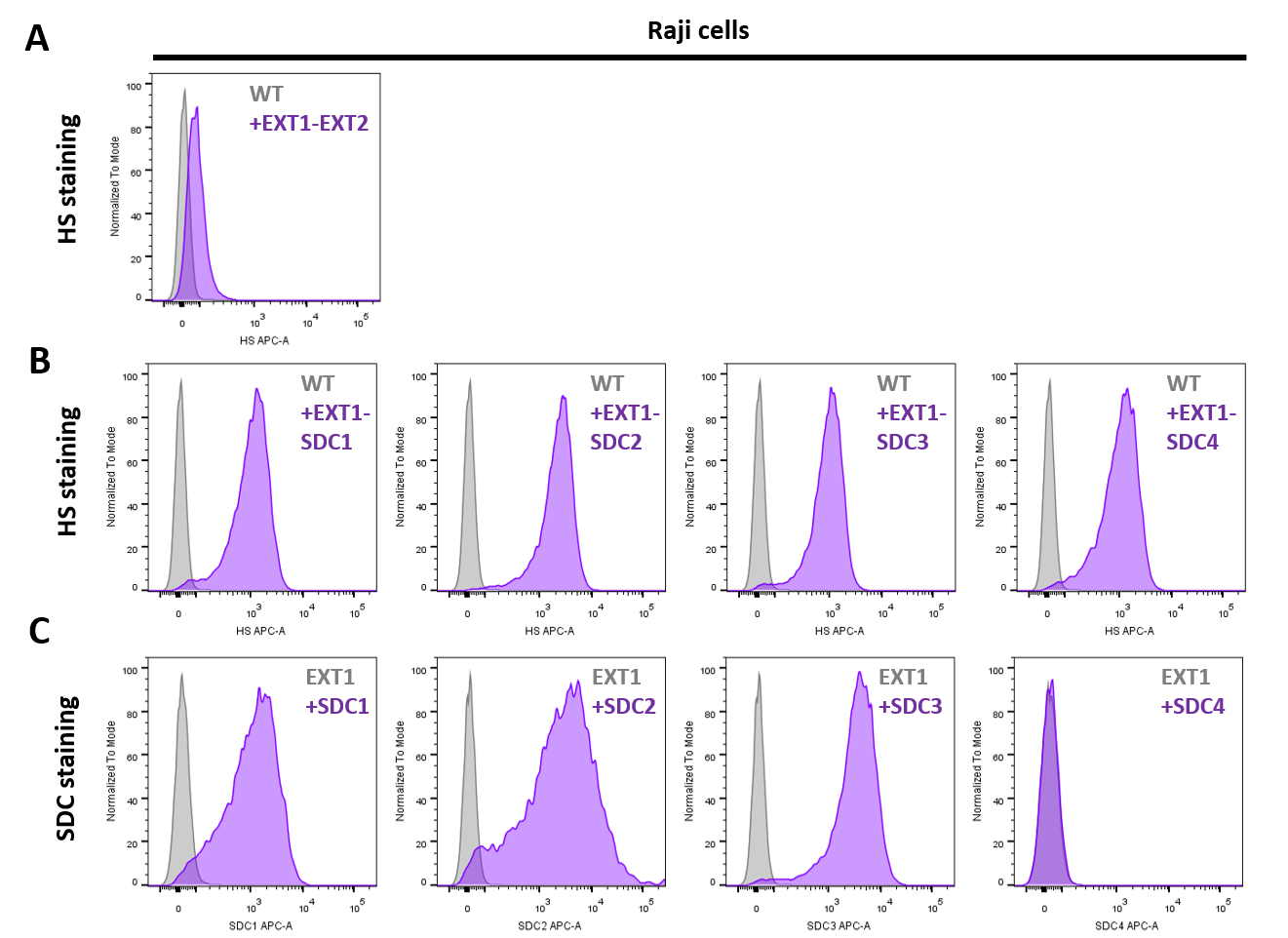

Supplement: S5 Fig — Flow cytometry analysis HS expression in Raji cells (A) stably expressing EXT1-EXT2 and (B) EXT1 + SDC1–4 stably transduced cells. (C) SDC staining of Raji-EXT1 cells stably transduced with SDC1–4. Cells were selected 48 h post transduction either with blasticidin (SDC vectors) or puromycin (EXT vectors). Marked in grey are either single transduced Raji cells (EXT1) or the WT. (TIF) [file ppat.1009996.s005.tif]
