# Supplementary material for: Heparan sulfate proteoglycans serve as alternative receptors for low affinity LCMV variants
Source: PLoS Pathog. 2021 Oct 14;17(10):e1009996. doi: 10.1371/journal.ppat.1009996 (PMC8547738; doi:10.1371/journal.ppat.1009996)
Supplement: S1 Table — (DOCX) [file ppat.1009996.s006.docx]

|  |  | HPI WT | WE HPI S153F | HPI high | Arm 53b | Arm Cl13 |
| --- | --- | --- | --- | --- | --- | --- |
|  | mutation | Y155H | S153F | H155Y | L260F | F260L |
|  | DAG1 affinity | Low | Low | High | Low | High |
| infection | 293T WT | +++ | +++ | +++ | +++ | +++ |
|  | 293T Δ*DAG1* | +++ | +++ | + | ++ | + |
|  | 293T Δ*EXTL3* | + | + | +++ | ++ | +++ |
|  | 293T Δ*DAG1 EXTL3* | - | - | - | - | - |
|  | 293T Δ*SLC35B2* | - |  | + | - | + |
|  | 293T Δ*DAG1 SLC35B2* | - |  | - | - | + |
| binding | 293T WT | +++ | +++ | +++ | +++ | +++ |
|  | 293T Δ*DAG1* | ++ | ++ | - | ++ | - |
|  | 293T Δ*EXTL3* | + | + | +++ | + | +++ |
|  | 293T Δ*DAG1 EXTL3* | - | + | - | - | - |
|  | Heparin-coated beads | Yes |  | Yes | Yes | Yes |
| Infection | Heparinase I/III | Inhib. |  | Inhib. (ΔDAG1) | Inhib. | Inhib. (ΔDAG1) |
|  | Heparin | Inhib. |  | Inhib. (ΔDAG1) | Inhib. | --- |
|  | Protamine sulfate | Inhib. |  | Inhib. (ΔDAG1) | Inhib. | Inhib. (ΔDAG1) |
|  | Chondroitin sulfate | --- |  | Inhib. (ΔDAG1) | --- | Inhib. (ΔDAG1) |
|  | Sodium chlorate | Inhib. |  | Inhib. (ΔDAG1) | Inhib. | Inhib. (ΔDAG1) |

**S1 Table. comparison of high and low affinity LCMV variants**
