# Supplementary material for: Heparan sulfate proteoglycans serve as alternative receptors for low affinity LCMV variants
Source: PLoS Pathog. 2021 Oct 14;17(10):e1009996. doi: 10.1371/journal.ppat.1009996 (PMC8547738; doi:10.1371/journal.ppat.1009996)
Supplement: S2 Table — (DOCX) [file ppat.1009996.s007.docx]

**S2 Table. human and murine CRISPR gRNAs**

| **Target** | **Species** | **gRNA name** | **Sequence** |
| --- | --- | --- | --- |
| *DAG1* | Human | DAG1 #1 | GATCTGCCTCCGGACGCGTT |
|  |  | DAG1 #2 | GAATAATGGCGCCTCGAGTC |
| *EXTL3* | Human | EXTL3 #1 | TGACTTGGTCGTATCACCGC |
|  |  | EXTL3 #2 | TGAACAACCGATTCTTACCC |
| *EXTL3* | Murine | EXTL3 #1 | GAGCACTTCCTCCCGCTCGT |
|  |  | EXTL3 #2 | ATTTCGGCCCATCGGTGGCG |
| *SLC35B2* | Human | SLC35B2 #1 | GCTGCGGGGCTCTGGTCCGC |
|  |  | SLC35B2 #2 | TTCTTCACACACCGGTCTCC |
