# Supplementary material for: Heparan sulfate proteoglycans serve as alternative receptors for low affinity LCMV variants
Source: PLoS Pathog. 2021 Oct 14;17(10):e1009996. doi: 10.1371/journal.ppat.1009996 (PMC8547738; doi:10.1371/journal.ppat.1009996)
Supplement: S3 Table — Italic sequence: overhang. (DOCX) [file ppat.1009996.s008.docx]

**S3 Table. Cloning primer for lentiviral transfer vector. Italic sequence: overhang**

| **Gene** | **For/Rev** | **Sequence** |
| --- | --- | --- |
| *EXT1* | for | *TATAGCTAGC*GACCCAGGCAGGACACATGC |
|  | rev | *TATATTAATTAA*TCCTCAAAGTCGCTCAATGTCTCGGT |
| *EXT2* | for | *TATAGCTAGC*ATGTCCTGCGCCTCAGGGTC |
|  | rev | *TATATTAATTAA*TCATAAGCTGCCAATGTTGGGGAAGC |
| *SDC1* | for | *TATAGCTAGC*ATGAGGCGCGCGGCGCTCTG |
|  | rev | *TATATTAATTAA*GGCTCCCGCGTCAGGCATAG |
| *SDC2* | for | *TATAGCTAGC*AATATGCGGCGCGCGTGGAT |
|  | rev | *TATATTAATTAA*TTACGCATAAAACTCCTTAGTAGGTGCC |
| *SDC3* | for | *TATAGCTAGC*ATGAAGCCGGGGCCGCCGC |
|  | rev | *TATATTAATTAA*GCACTGTGGCTCCACTAGGCATAG |
| *SDC4* | for | *TATAGCTAGC*ATGGCCCCCGCCCGTCTGTTC |
|  | rev | *TATATTAATTAA*GCTTCACGCGTAGAACTCATTGGTGG |
| *GPC1* | for | *TATAGCTAGC*ATGGAGCTCCGGGCCCGAG |
|  | rev | *TATATTAATTAA*TTACCGCCACCGGGGCCTG |
| *Hygromycin* | for | *ATTGTCGACTCCGCCGCGGCCGCGC*CGAAATCTCGTAGCACGTGC |
|  | rev | *GATAAGCTTGCCACAACCACCGGGA*TCCATTTTCGGATCTGATCAGC |
| *Puromycin* | for | *CGATGATAAGCTTGCCACAACCACCGGGAT*ATGACCGAGTACAAGCCCACGG |
|  | rev | *GAGGTTGATTGTCGACTCCGCCGCGGCCGC*TCAGGCACCGGGCTTGCGGG |
