# Supplementary material for: Heparan sulfate proteoglycans serve as alternative receptors for low affinity LCMV variants
Source: PLoS Pathog. 2021 Oct 14;17(10):e1009996. doi: 10.1371/journal.ppat.1009996 (PMC8547738; doi:10.1371/journal.ppat.1009996)
Supplement: S4 Table — (DOCX) [file ppat.1009996.s009.docx]

**S4 Table. Antibodies for Flow cytometry analysis**

| **Target** | **Species** | **Dilution** | **Catalog Nb.** | **Company** |
| --- | --- | --- | --- | --- |
| HS (10E4) | human | 1:100 | 370255-S | Amsbio |
| SDC4 | human | 1:100 | MAB29181 | R&D Systems |
| αDAG1 (IIH6) - 488 | human | 1:100 | Sc-53987 | Santa Cruz Biotechnology |
| SDC2 - APC | human | 1:100 | 130-107-534 | MACS Miltenyi Biotec |
| SDC3 - APC | human | 1:100 | FAB3539A | R&D Systems |
| SDC1 - APC (DL-101) | human | 1:100 | 352307 | BioLegend |
| SDC4 - APC | mouse | 1:50 | 130-109-831 | MACS Miltenyi Biotec |
| IgM (II/41) | mouse | 1:250 | 550676 | BD Biosciences |
| CD8a-FITC | mouse | 1:200 | 553031 | BD Biosciences |
| LCMV N (VL-4) | LCMV | 1:100 | BE0106 | InVivoMab |
| IgG - FITC | rat | 1:500 | A18740 | Invitrogen |
