# Supplementary material for: Heparan sulfate proteoglycans serve as alternative receptors for low affinity LCMV variants
Source: PLoS Pathog. 2021 Oct 14;17(10):e1009996. doi: 10.1371/journal.ppat.1009996 (PMC8547738; doi:10.1371/journal.ppat.1009996)
Supplement: S5 Table — (DOCX) [file ppat.1009996.s010.docx]

**S5 Table. NGS primer for nested PCR**

| **Name** | **Illumina P5 or P7** | **barcode** | **Stagger** | **Priming site** |
| --- | --- | --- | --- | --- |
| for NGS 1st |  |  |  | TAGTGAATAGAGTTAGGCAGG |
| rev NGS 1st |  |  |  | TAGCGAATTGTGGATGAATACTGCC |
| pLenti_F1 | AATGATACGGCGACCACCGAGATCTACACTCTTTCCCTACACGACGCTCTTCCGATCT | CTGACT |  | GTGGAAAGGACGAAACACCG |
| pLenti_F2 |  | ACTGAC | N |  |
| pLenti_F3 |  | TGACTG | NN |  |
| pLenti_F4 |  | GACTGA | NNN |  |
| pLenti_F5 |  | CTAGCA | NNNN |  |
| pLenti_F6 |  | ACGTAG | NNNNN |  |
| pLenti_F7 |  | TGCATC | NNNNNN |  |
| pLenti_F8 |  | GATCGT |  |  |
| pLenti_F9 |  | CGTACG | N |  |
| pLenti_F10 |  | ATCGAT | NN |  |
| pLenti_F15 |  | GTACGG |  |  |
| pLenti_R univ | CAAGCAGAAGACGGCATACGAGATGTGACTGGAGTTCAGACGTGTGCTCTTCCGATCT |  |  | GCGAATTGTGGATGAATACTGCC |
